# Genome Mining Guided Identification of the Metallophore Delftichelin A from *Delftia deserti*

**DOI:** 10.1021/acs.jnatprod.6c00213

**Published:** 2026-03-27

**Authors:** Martinus de Kruijff, Lukas Hiller, Joy Birkelbach, Tanya Decker, Sebastian Götze, Rebecca Kochems, Rolf Müller, Anna K. H. Hirsch, Christine Beemelmanns

**Affiliations:** † 443745 Helmholtz Institute for Pharmaceutical Research Saarland (HIPS)—Helmholtz Centre for Infection Research (HZI), 66123 Saarbrücken, Germany; ‡ Department of Pharmacy, Saarland University, 66123 Saarbrücken, Germany; § Faculty of Medicine, Saarland University, 66123 Saarbrücken, Germany

## Abstract

A targeted sequencing and genome mining approach for delftibactin-like biosynthetic pathways revealed three distinct biosynthetic gene cluster architectures (BGC *del*, *dlc* and *dlp*) encoded in genomes of members of the genus *Delftia*. Comparative metabolomic analysis guided the isolation and characterization of a yet unreported metallophore, delftichelin A from *Delftia deserti* DSM1621 (previously named *Delftia acidovorans* DSM1621). Prediction of BGC architecture and A domain specificity was in line with the structure analysis uncovering previously unreported differences in amino acid composition and modifications. Analysis of bioactivity and metal-binding characteristics demonstrated that delftichelin A shows a preferential affinity for ferric iron, while also exhibiting heavy metal detoxification mechanisms via oxidative degradation, analogous to those reported for the delftibactin family of compounds.

## Graphical abstract

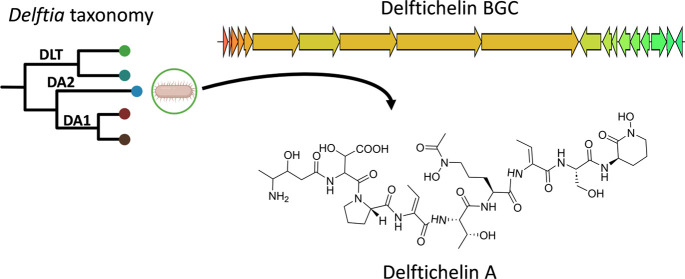

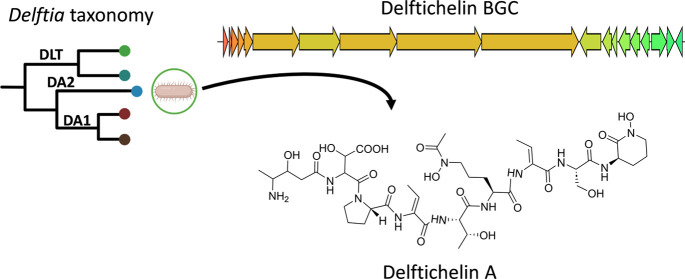

Virtually all microorganisms are in constant need for iron as an essential nutrient for growth.^1^ However, the bioavailability of ferric iron (Fe­(III)) is exceedingly limited due to its predominant occurrence in its highly insoluble form, ferric oxide.^2^ To satisfy sufficient iron availability, microorganisms have evolved the capability to produce specialized natural products with metal ion chelating properties of low molecular weight (typically 500–1500 Da), also termed siderophores.^3–6^ While iron binding is often the primary function of many metallophores, some preferentially chelate other metal ions based on their structural features, exhibiting high affinities for divalent species such as Ni­(II) and Cu­(II), as well as trivalent ions such as Co­(III), Mn­(III) and even Au­(III).^7,8^ The metal-chelating properties and binding affinities of bacterial metallophores are governed by the type and number of their functional groups, as well as the length of the connecting linker. Structural coordination motifs such as catecholates, hydroxamates, and α-hydroxycarboxylates typically serve as bidentate *O*,*O*-donor metal-binding groups.^6,9^ More often the rule than the exception, additional chelating moieties such as β-hydroxyaspartic acid and *N*(δ)-hydroxyornithine are also sometimes part of metallophore structures likely to address the needs of metal-specific coordination geometries to enable the formation of stable coordination complexes with metal ions. Prominent examples are loihichelins A-F^10^, and potashchelins A–D^11^. Another example is delftibactin A (**1**) (Figure 1), an unqiue bacterial metallophore that allows *Delftia acidovorans* to survive metal stress from toxic gold ions.^12,13^ Delftibactins contains among other features, β-hydroxyaspartic acid and *N*(δ)-hydroxy-ornithine moieties.^14,15^ Upon interaction with Au­(III) ions, delftibactin A (**1**) leads to a stepwise reduction of Au­(III) ions to inert gold nanoparticles by an intrinsic reduction process, which leads to an oxidative degradation of the metallophore.^16^

**Figure 1:**
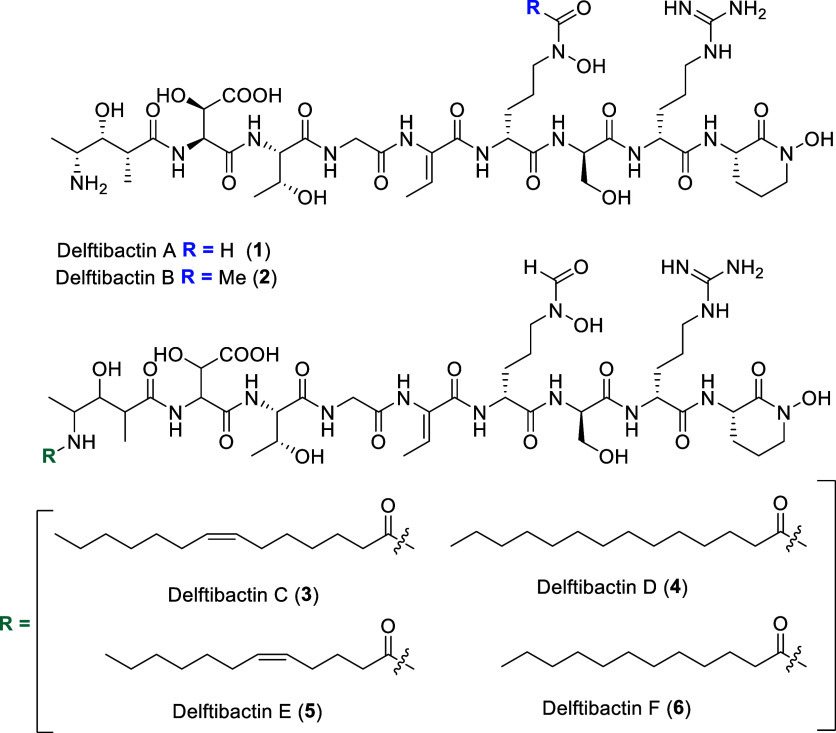
Known chemical structures of delftibactins A-F.

Given the crucial role of metallophores in metal bioremediation across diverse environments^17^ and driven by our pursuit of discovering biosynthetic diversity, we employed a genome mining approach on selected reference genomes of *Delftia* spp. due to their promising potential for producing novel metal-chelating natural products. Part of this investigation revealed a previously unreported biosynthetic gene cluster (BGC) that shares partial homology with the delftibactin BGC identified in *D. acidovorans* DSM 1621, and other *Delftia* isolates.^14^ Intrigued by the divergent gene cluster architecture of the delftibactin BGC, but limited by the available genomic data of selected DSMZ strains for further comparative BGC analyses, we combined a long-read sequencing approach with high-resolution metabolomics. This approach led to the discovery of a new group of delftibactin-like metallophores, named delftichelins, and the subsequent evaluation of bioactivity and metal-binding properties of the major representative, delftichelin A (**7**).

## Results and Discussion

### Targeted Genome Mining for Delftibactin-like Biosynthesis

In light of the promising capacity of *Delftia* spp. to produce metallophores, we undertook a genome mining approach using the four *Delftia* reference genomes available in the NCBI database (*D. acidovorans* SPH-1, *D. deserti* KCTC 42377, *Delftia lacustris* DSM 21246 and *Delftia tsuruhatensis* ULwDis3). In addition, *D. acidovorans* DSM39 was included as it had previously been reported to produce delftibactins and was available from the DSMZ culture collection. This strain, along with a second DSMZ strain of the same species (*D. acidovorans* DSM1621), was purchased and subsequently genome-sequenced to expand the data set used for genome mining analysis (Table S1).^14,18^

We first surveyed the overall biosynthetic potential using the “antibiotics and secondary metabolite analysis shell” (antiSMASH, v8)^19^ and “Biosynthetic Gene Similarity Clustering and Prospecting Engine” (BiG-SCAPE, v2.0)^20^ with default settings. Identified BGCs were compared to characterized clusters of the “Minimum Information about a Biosynthetic Gene cluster” (MIBiG) database (v4.0).^21^ Intriguingly, the overall biosynthetic potential ranged from five complete BGCs observed for *D. acidovorans* SPH-1 reaching up to ten for *D. acidovorans* DSM1621 (Figure 2A, Tables S1 and S2). The BGC types encoded by *D. acidovorans* DSM1621 differed from *D. deserti* KCTC42377 only by a single BGC encoding butyrolactone biosynthesis, which was present in DSM1621 but absent in KCTC42377. Importantly, all six *Delftia* strains possessed a NRPS/PKS-hybrid metallophore BGC that was assigned to delftibactin-like biosynthesis. Subsequently, we analyzed the boundaries and architecture of the NRPS/PKS-hybrid BGCs in more detail using cLINKER (v0.0.31)^22^ for pairwise similarity analysis (Figure 2B).

**Figure 2:**
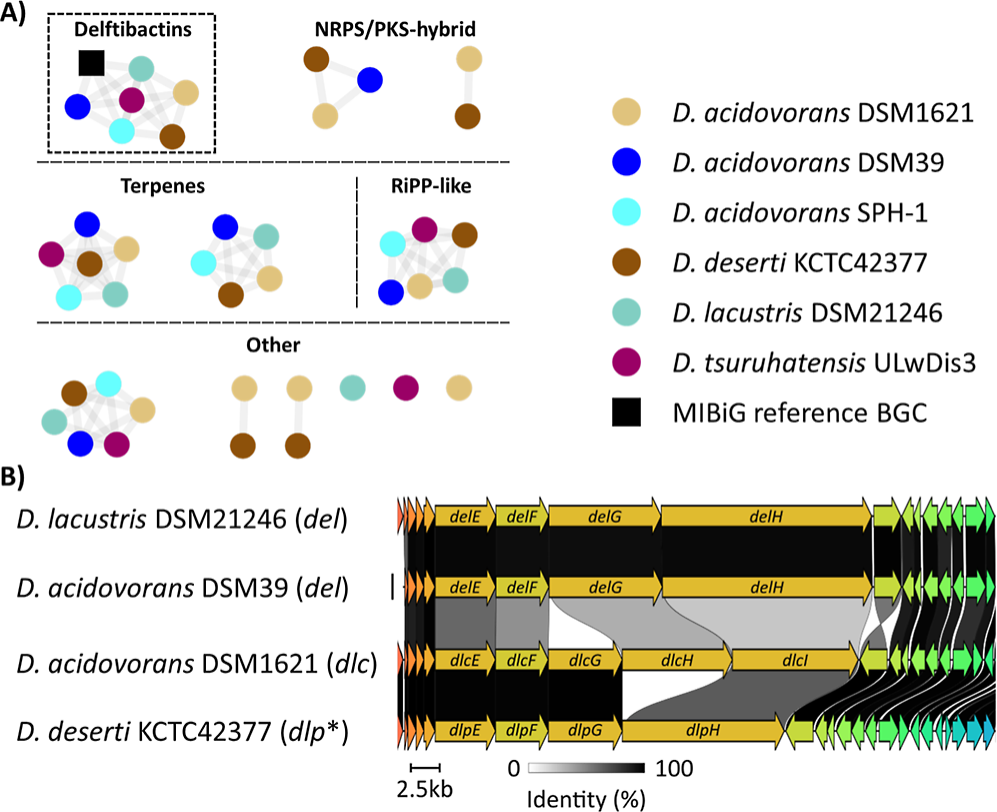
Genome mining of *Delftia* strains for BGCs encoding secondary metabolites. (A) BiG-SCAPE network with a subnetwork containing homologues of delftibactin-like BGCs. (B) cLINKER comparison of core biosynthetic genes of delftibactin-like BGCs (Figure S1). Gene similarities are indicated by links with a grey scale from 0% (white) to 100% (black) identity. Genes are colored according to similarities >30%, while gray indicates similarities below 30% identity. The product of dlp* (*D. deserti* KCTC42377) is unknown.

The detailed comparison revealed an overall good to high sequence similarity across the five BGCs but with important variations within the gene arrangements and notable differences for some of the core NRPS and PKS genes. Here it needs to be noted that the BGCs encoded by *D. acidovorans* DSM39 and *D. lacustris* DSM21246 showed similarities above 95%, and thus were both annotated as delfitibactin BGCs (*del*). Moreover, *D. acidovorans* SPH-1 and *D. tsuruhatensis* ULwDis3 were omitted from the alignment due to their almost identical BGCs to *D. lacustris* DSM21246. In contrast, two BGCs encoded by the taxonomically closely related *D. deserti* KCTC42377 and *D. acidovorans* DSM1621, respectively, showed specific gene cluster differences to *del* and each other. We therefore assigned the BGC from *D. deserti* KCTC42377 as a *dlp* BGC encoding a yet uncharacterized delftibactin-like natural product. Based on differences in gene cluster architecture and predicted amino acid incorporation, substantial structural divergence from the reported delftibactins (*del*) is anticipated and thus we tentatively named the yet cryptic product delftipeptin (Figure S2).

The BGC retrieved from *D. acidovorans* DSM1621 (hereafter *dlc*) shows also notable differences in several in core NRPS and PKS gene compared to the delftibactin (*del*) and delftibactin-like (*dlp*) BGCs. In particular, the core NRPS and PKS genes of *dlc* share only low sequence similarity (33–67%) with those of *del* (Figures S1 and 2B). Contrary, *dlc* and *dlp* shared high sequence similarities of 99–100%. However, *dlcH* and *dlcI* appear to be fused and truncated into *dlpH*, a single gene that lacks the sequence information for two C-A-T modules compared to *delH* (Figure S1). More detailed comparative analysis of the *dlc* revealed distinct differences in module organization, predicted substrate specificities, and the incorporation of an additional NRPS-encoding gene. Specifically, in contrast to *del*, the *dlc* cluster lacks modules predicted to incorporate glycine or arginine. Instead, modules corresponding to proline incorporation (*dlcG*) and an additional threonine-incorporating module (*dlcI*) were identified. Overall, our comparative analysis of the BGCs substantiated the presence of architecturally divergent delftibactin-like BGCs, supporting the likelihood that these clusters encode for the biosynthesis of structurally distinct metallophore variants.

### Taxonomy

Due to ambiguities within the taxonomic positioning for some of the *Delftia* isolates,^23^ as well as the notable similarity between the BGC composition of *D. acidovorans* DSM1621 and *D. deserti* KCTC42377, we performed whole genome-based phylogenomic analysis (Figure S3).^24^

An abridged taxonomic tree analysis (Figure 3) showed three main clade distinctions, with *D. deserti* KCTC42377 and *D. acidovorans* DSM1621 forming one clade (DA2) that separates the *D. tsuruhatensis* (clade DLT) and *D. acidovorans* strains (clade DA), similar to the previously reported formation of different clades for this genus.^23,25^ It was thus noted that *D. acidovorans* DSM1621 (also referred to as *D. acidovorans* FDAARGOS909) shared a gene support index (GI) of 100% and formed a clade with *D. deserti* KCTC42377 (ANIm: 99.24%, ddH: 87.7%) with a GI of 88% (Figure S3), while no grouping with other *D. acidovorans* strains was observed. Based on these findings, we propose with high confidence that *D. acidovorans* DSM1621 should be reclassified as *D. deserti* DSM1621 and will hereafter be referred to as such. Moreover, the delftibactin-like BGCs were detected in the genomes from three distinct phylogenomic clades of the *Delftia* genus, and detected in strains isolated from distinct environmental sources.

**Figure 3:**
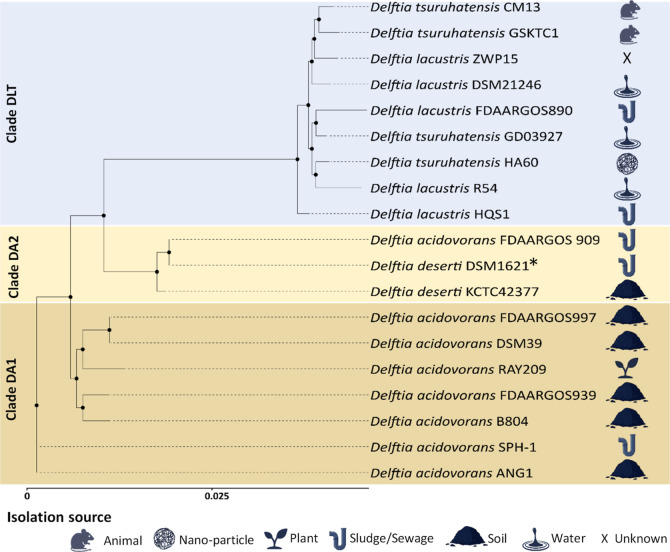
Unrooted phylogenomic tree inferred using a concatenated alignment of 81 core genes of selected species from the genus *Delftia*. *D. deserti* DSM1621, marked with an asterisk (*), was previously classified as *D. acidovorans* DSM1621. Isolation sources were displayed according to Bhat et al.^23^ Isolation source symbols were created in BioRender.com.

### Metabolomic Analysis and Structure Elucidation

Due to the observed differences in BGC architectures and the hypothesized structural differences to be expected for their natural products, we then aimed to provide a proof of principle by isolation of the predicted chemical scaffolds. As siderophore biosynthesis is often tightly regulated and induced only in response to specific environmental cues,^26^ we first examined the BGCs (*dlc*, *dlp*) for the presence of putative transcriptional regulators. Two sigma-70 family RNA polymerase sigma factor regulators were annotated, one upstream and one downstream of the core biosynthetic genes. Due to the high sequence similarity to regulators also encoded in the *del* BGC, we speculated that iron-deficient conditions should influence the BGC transcription, similar to prior studies on delftibactin.^14,15,18^ To identify potential products of *dlc* and *dlp*, *D. deserti* DSM1621, *D. deserti* KCTC42377 and for comparison *D. acidovorans* DSM39, known to produce delftibactins, were cultivated in “Defined Medium for Siderophores” (DMS)^27^ under both, iron-deficient (no additives) and iron-replete (supplemented with 3 μM ferric citrate) conditions.

After 3 days of cultivation at 200 mL scale, the supernatants were extracted via solid phase extraction (C18-SPE) and eluted with 50% and 100% acetonitrile (ACN) in water (H_2_O). Notably, a high abundance of ions was present in the total ion chromatogram (TIC) at about 2.85 min in the iron-deficient 50% ACN extract of *D. deserti* DSM1621, that were absent from the iron-replete control (Figure S4, for respective extracted ion chromatograms see Figure 4A). Under iron-depleted conditions, *D. deserti* KCTC42377 did not produce different compounds compared to the iron-replete control. We then processed the data of *D. acidovorans* DSM39 and *D. deserti* DSM1621 using MZmine and compared molecule consensus features using the “feature based molecular networking” (FBMN)^28,29^ function of Global Natural Products Social Molecular Networking (GNPS)^29^ and data was visualized in Cytoscape^30^ (Figure 4B).

**Figure 4:**
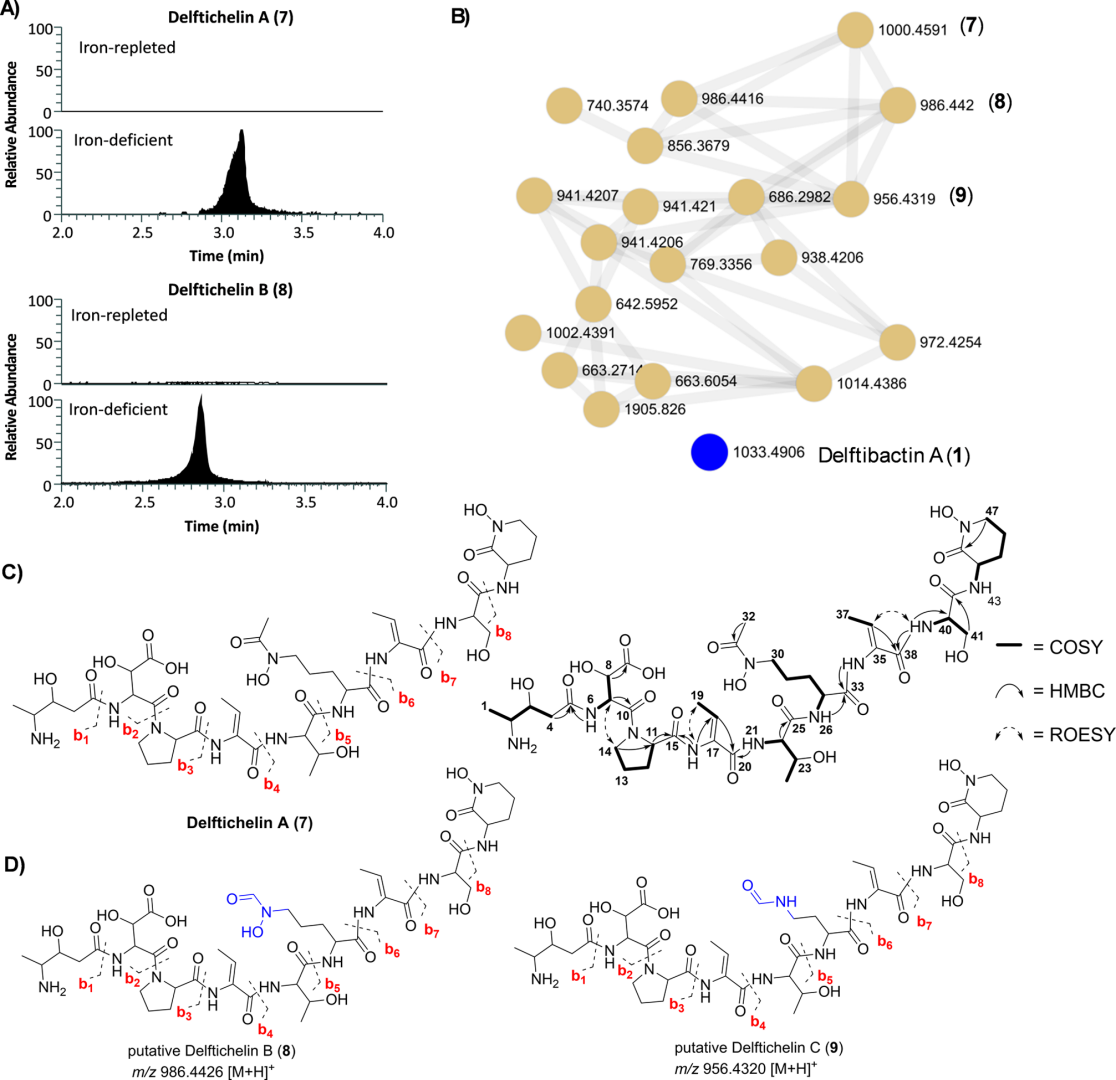
Metabolomic comparison of *D. deserti* DSM1621 with *D. acidovorans* DSM39 and chemical structures of delftichelin derivatives. (A) Extracted ion chromatograms (EICs) of *m*/*z* features assigned to delftichelin within 50% ACN extracts of *D. deserti* DSM1621 grown in iron-replete (3 μM iron­(III) citrate) and iron-deficient conditions. EICs were normalized (relative abundance) and smoothed using the “Moving Mean” algorithm. (B) A GNPS subnetwork of delftichelin-like metabolites produced by *D. deserti* DSM1621 (light brown nodes), alongside delftibactin A (**1**), which appears as a single node produced by*D. acidovorans* DSM39 (blue node) under iron-deficient conditions. (C) Observed b-ions within HRMS/MS fragmentation of delftichelin A (**7**) (Table S3) and key COSY, ROESY and HMBC correlations observed during NMR analysis of delftichelin A (**7**). D) Putative structures of delftichelin B (**8**) and C (**9**) based on HRMS/MS fragmentation (Tables S3 and S4, Figures S6 and S7).

Dereplication of metabolites within the GNPS network for strains DSM39 and DSM1621 grown under iron-repleted conditions revealed, as expected, the absence of known metallophores, with the sole exception of *D. acidovorans* DSM39, which produced delftibactin A (**1**) (Figure 4B). Intriguingly, the metabolome of *D. deserti* DSM1621 (*dlc* BGC) under iron-deficient conditions shifted to produce another molecular subnetwork within a similar mass range encompassing unknown features (e.g., *m*/*z* 1000.4591 [M + H]^+^). Similarly to delftibactin A (**1**), the unknown metabolites detected were induced by iron-starvation, suggesting siderophore functionality. When compared against publicly available databases such as GNPS, the Natural Products Atlas (NPAtlas),^31^ and the COlleCtion of Open NatUral ProducTs (COCONUT),^32^ no matches were found for the main *m*/*z* 1000.4591 [M + H]^+^. However, putative HRMS/MS fragmentation data (b-ions listed in Table S3; Figure S5) suggested the presence of various proteinogenic and nonproteinogenic amino acids, consistent with the amino acid composition in the *dlc* BGC predicted by antiSMASH. This molecular feature is hereafter designated as delftichelin A (**7**). Further inspection of related mass features within the subnetwork allowed the prediction of two additional, putative congeners *m*/*z* 986.442 [M + H]^+^ and *m*/*z* 956.4319 [M + H]^+^ designated as delftichelin B (**8**) and delftichelin C (**9**), based on HRMS/MS fragmentation data (Figure 4D, respective b-ions listed in Tables S3 and S4, Figures S6 and S7).

Thus, we upscaled production under iron-deficient conditions (2 L total) and purified the metabolites from ACN extracts using a MS- and activity-guided approach. Following the bioactivity in the chrome azurol S (CAS)-assay relating to siderophore presence, the 50% ACN solid phase extraction fraction was further fractionated by HPLC (C18 semipreparative column, Figure S8) yielding one fraction showing strong CAS-activity (Figure S8). While low production titers and the challenging highly polar purification processes prevented the isolation of congeners delftichelin B (**8**) and delftichelin C (**9**) in sufficient quantities, we were able to obtain delftichelin A (**7**, [M + H]^+^ 1000.4591 *m*/*z*, molecular formula C_41_H_65_N_11_O_18_) in analytical purity.

### Deduction of Planar Chemical Structure of Delftichelin A

In alignment to previously published delftibactin,^14^ HRMS/MS fragmentation analysis of delftichelin A (Figures S5 and S9) revealed an *N*-terminally modified octapeptide consisting of proteinogenic amino acids threonine and serine, and nonproteinogenic or modified amino acids β-OH-aspartic acid (β-OH-Asp), dehydrobutyrine (Dhb), *N* ^δ^-OH-*N* ^δ^-acetyl-ornithine (*N* ^δ^-OH-*N* ^δ^-acetyl-Orn) and cyclic *N* ^δ^-OH-ornithine (*N* ^δ^-OH-Orn). As expected from our BGC analysis, the overall amino acid sequence, the *N*-terminal residue and two amino acids did not align with delftibactin, and were subsequently elucidated using comprehensive Nuclear Magnetic Resonance spectroscopy (NMR) analysis (Figures 4C and S10–S15, Table 1). In particular, characteristic HSQC cross peaks, as well as COSY and HMBC correlations revealed proline (Pro) and an additional Dhb as the two elusive amino acids. Analysis of the vicinal coupling constants in the Dhb moieties led to the assignment of a *Z*-configuration in both cases (*J*_18,19_ 7.1 Hz, *J*_36,37_ 7.1 Hz). Moreover, the *N*-terminal residue of delftichelin was identified as 4-amino-3-hydroxypentanoic acid, which, in contrast to the characteristic methylated methine group in the *N*-terminal side chain of delftibactin, contains a diastereotopic methylene group. The structure was deduced from the close similarity of proton and carbon chemical shifts, supported by COSY and HMBC correlations involving CH_3_-1, CH-2, and CH-3. Finally, the complete amino acid sequence was established through a combination of HRMS/MS fragmentation analysis, which was in line with detectable HMBC and ROESY correlations and bioinformatic considerations (Figure 4C).

**Table 1:**
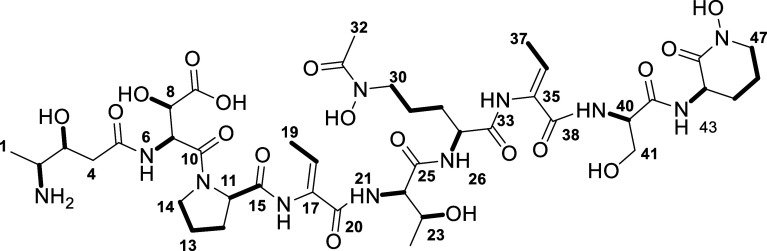
^1^H (700 MHz) and ^13^C (150 MHz) NMR Spectroscopy Data for Delftichelin A (DMSO-*d*_6_)

residue	Pos	δ_C_, X type	δ_H_, (*J* in Hz)	COSY	HMBC^a^	ROESY
Ahpa	1	12.7, CH_3_	1.08, d (6.7)	2	2, 3	2
	2	50.0, CH	3.15, m	1, 3	1, 3	1, 3
	2-NH	-, NH	n.d	-	-	-
	3	67.9, CH	4.00, m	2, 4ab	1, 2, 4	2
	3-OH	-, OH	n.d	-	-	-
	4a	39.5, CH_2_	2.33, dd (14.5, 7.5)	3	2, 3, 5	6
	4b		2.38, dd (14.5, 5.8)	3	2, 3, 5	6
	5	169.7, C	-	-	-	-
β-OH-Asp	6	-, NH	7.90, br d (7.2)	7	5	4ab, 8
	7	53.6, CH	4.82, br dd (7.2, 4.7)	6, 8	8, 9, 10	8, 14a
	8	72.0, CH	3.95, br d (4.3)	7	7, 9, 10	6, 7
	8-OH	-, OH	n.d	-	-	-
	9	174.3, C	-	-	-	-
	9-OH	-, OH	5.41, br s	-	-	-
	10	169.8, C	-	-	-	-
Pro	11	60.7, CH	4.38, br dd (8.3, 4.9)	12ab	12, 13, 14, 15	12b, 16
	12a	29.0, CH_2_	1.99, m	11, 12b	13, 14	-
	12b		2.10, dq (12.3, 7.9)	11, 12a, 13	11, 13, 14, 15	11
	13	24.5, CH_2_	1.90, m	12, 14ab	11, 12, 14	-
	14a	47.5, CH_2_	3.64, m	13, 14b	11, 12, 13	7, 14b
	14b		3.92, m	13, 14a	12, 13	-
	15	170.8, C	-	-	-	-
Dhb-1	16	-, NH	9.24, br s	19	15, 18	11, 19
	17	129.8, C	-	-	-	-
	18	130.6, CH	6.50, q (7.1)	19	17, 19, 20	19
	19	13.2, CH_3_	1.61, m	16, 18	17, 18, 20	16, 18
	20	164.2, C	-	-	-	-
Thr	21	-, NH	7.50, br d (7.8)	22	20, 22	22, 23
	22	59.3, CH	4.12, br d (2.8)	21	23, 25	21, 23-OH, 24
	23	66.1, CH	4.10, m	24	25	21, 24
	23-OH	-, OH	9.07, m	-	25	22
	24	20.0, CH_3_	1.05, m	23	22, 23	22, 23
	25	171.2, C	-	-	-	-
*N* ^δ^-OH-*N* ^δ^-acetyl-Orn	26	-, NH	7.96, br d (8.1)	27	27, 33	27, 28a
	27	49.8, CH	4.27, m	26, 28ab	28, 29	26, 28b, 29a
	28a	27.4, CH_2_	1.65, m	27, 28b	27, 30	26
	28b		1.87, m	27, 28a	30	27
	29a	20.1, CH_2_	1.82, m	30	27	27
	29b		1.89, m	30	27	30
	30	51.2, CH_2_	3.46, m	29ab	29	29b
	30-N–OH	-, N–OH	n.d	-	-	-
	31	170.3, C	-	-	-	-
	32	20.4, CH_3_	1.96, s	-	31	-
	33	164.5, C	-	-	-	-
Dhb-2	34	-, NH	4.26, m	-	33	-
	35	130.4, C	-	-	-	-
	36	127.9, CH	6.34, q (7.1)	37	35, 37, 38	37, 39
	37	12.8, CH_3_	1.61, m	36	35, 36, 38	36
	38	164.0, C	-	-	-	-
Ser	39	-, NH	7.54, br d (7.8)	40	38, 40, 41	36, 40, 41
	40	55.9, CH	4.27, m	39, 41	38, 41, 42	39, 41
	41	61.7, CH_2_	3.63, m	40	40, 42	39, 40
	41-OH	-, OH	n.d	-	-	-
	42	169.6, C	-	-	-	-
cyclic *N* ^δ^-OH-Orn	43	-, NH	8.40, br s	44	-	44
	44	53.9, CH	4.13, br s	43, 45	45, 46	43
	45	27.7, CH_2_	1.68, m	44	44, 46	-
	46a	23.2, CH_2_	1.53, m	-	47	-
	46b		1.61, m	-	47	-
	47a	46.6, CH_2_	3.42, m	-	45, 46	-
	47b		3.52, m	-	45, 46, 48	-
	47-N–OH	-, N-OH	n.d	-	-	-
	48	170.3, C	-	-	-	-

a Protons showing HMBC correlations to listed carbons. n.d. Not detected.

Based on HRMS/MS data, delftichelin B (**8**) differed from delftichelin A (**7**) by incorporation of *N* ^δ^-formyl-*N* ^δ^-hydroxy-ornithine instead of *N* ^δ^-acetyl-*N* ^δ^-hydroxy-ornithine, while delftichelin C (**9**) differed from delftichelin B (**8**) by incorporation of *N* ^δ^-formyl-2,4-diaminobutyric acid (DAB) instead of *N* ^δ^-formyl-*N* ^δ^-hydroxy-ornithine (Figure 4D).

### Biosynthetic Analysis and Determination of Amino Acid Configuration

To determine the configuration of the amino acids, we combined Marfey’s analysis with biosynthetic pathway analyses.^33^ Acid-catalyzed hydrolysis followed by derivatization with l-FDVA identified l-proline, l-threonine, d- and l-ornithine, and l-serine (Figures S16–S20). However, despite applying various hydrolysis conditions, the configuration of β-hydroxyaspartic acid (3-OH-Asp) could not be determined, likely due to degradation processes during hydrolysis similar to those reported for delftibactins, as both delftibactin and delftichelin undergo distinct decomposition under these conditions.^18^

Subsequently, we revisited the predicted domain and module architecture of the encoding BGC (Figure 5). Despite the overall moderate to high similarities of the BGCs *dlc* and *del*, the following chemical product differences were observed and correlated to the respective differences in the pathways. Strikingly, the antiSMASH (v7.1 and v8) predicted NRPS/PKS architectures of *dlc* (Ser-Mal-Asp-Pro-Thr-Thr-Formyl-Hydroxy-Orn-Thr-Ser-Hydroxy-Orn) and *del* (Ser-Mal-Asp-Thr-Gly-Thr- Orn-Ser-Arg-Hydroxy-Orn) differ notably and the most prominent distinctions are the incorporation of proline, as well as the absence of arginine and glycine in delftichelin A (**7**) (Figure S9).

**Figure 5:**
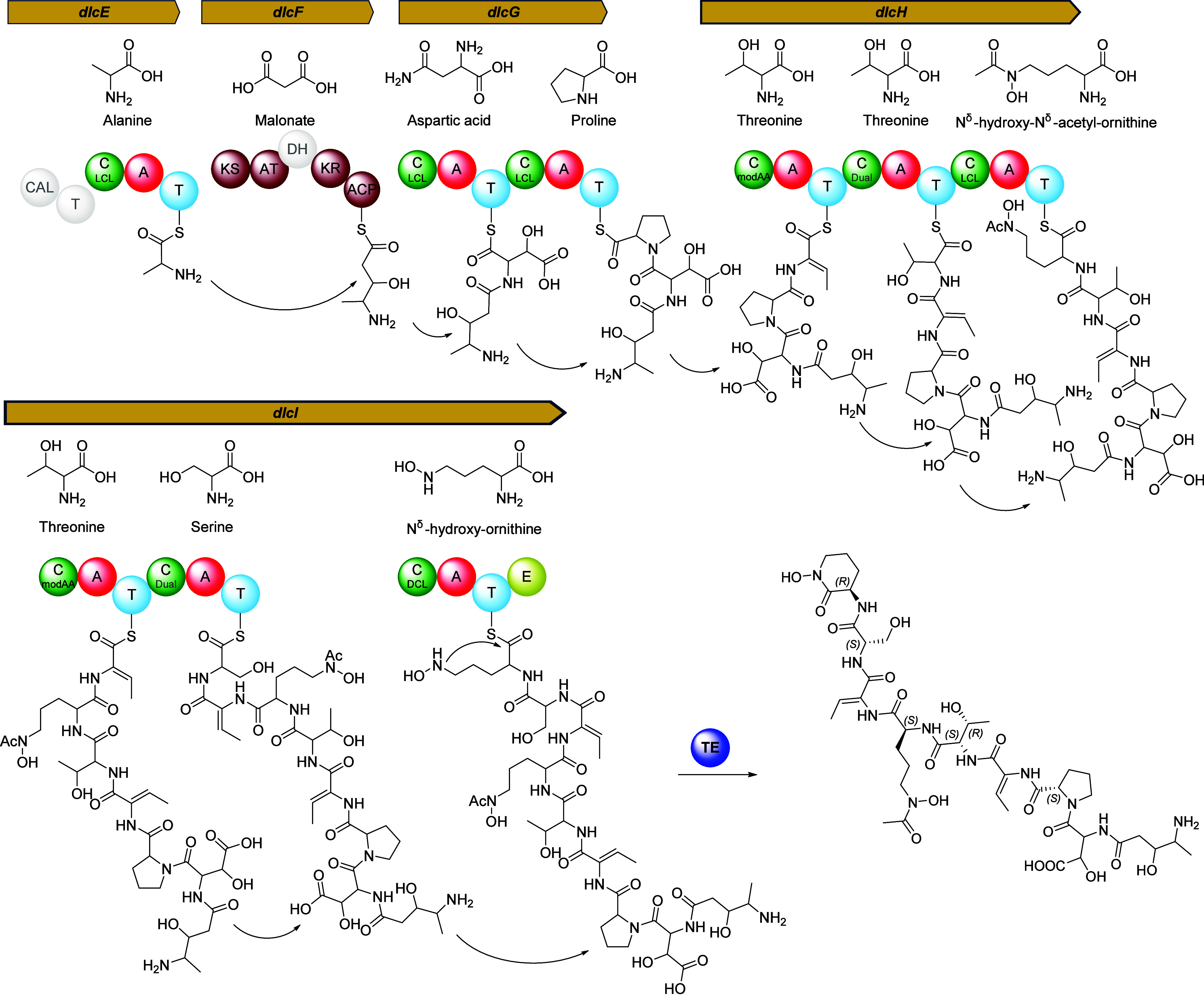
Proposed biosynthesis pathway of delftichelin A. Large arrows depict the biosynthesis genes in relative scales. A-domain substrates are depicted above their respective domains. Functions of subdomains within C-domains are illustrated, while inactive subdomains are shown in gray. Light gray domains represent predicted inactive domains. Abbreviations: A = adenylation domain, ACP = acyl carrier protein, AT = acyltransferase domain, C = condensation domain, CAL = coenzyme A ligase, DH = dehydratase, E = epimerase domain, KR = ketoreductase, KS = ketosynthase domain, T = thiolation domain, TE = thioesterase.

By taking into account the presence of epimerization domains and the substrate specificities of the A-domains,^14,18^ we then inferred the likely configurations of the amino acids. Starting with the *N-*terminus side chain, the relatively high identity of DlcE and DelE (67%) let us assume that l-alanine is the most preferred substrate of the NRPS-PKS hybrid, incorporating l-alanine over l-serine similar to delftibactin A (**1**). The elongating PKS modules DelF and DlcF contain a DH domain, which presumably is inactive as no product with an unsaturated carbon–carbon bond was identified in delftichelin nor was reported previously for delftibactins. Also, similar to delftibactins, the configuration of the [4-amino-3hydroxy-pentanoic acid] headgroup biosynthesis was not predictable.^14,18^ However, due to the similarity of DelF and DlcF (55% identity), the configuration is proposed to be similar to delftibactin A (**1**), which would suggest (3*R*,4*S*) [4-amino-3hydroxy-pentanoic acid].^16^ Notably, methyl-malonyl is incorporated in delftibactins, whereas malonyl is incorporated into delftichelins, consistent with the predicted substrate specificity of the AT-domains of DelF and DlcF.

Furthermore, we hypothesize that 3-OH-Asp occurs in l-erythro configuration given the lack of an epimerization domain in DlcG based on the moderate similarities to DelG (52% with a C-domain similarity of 57%).^16,34^ Due to the very high sequence identity of DlcD and DelD (99%), it is reasonable to assume that the stand-alone β-hydroxylase DlcD acts on the aspartic acid on the tethered chain resulting in a (2*S*,3*R*) configuration, as previously reported.^16,34^

As predicted, proline was incorporated into delftichelin A (**7**) in l-configuration (Figure S17). The second threonine incorporated by DlcH was bioinformatically predicted to be incorporated as d-threonine, based on the dual-condensation domain exhibiting both condensation and epimerization functions, but l-threonine was observed (Figure S18) indicating an inactive epimerization subdomain of DlcH. Moreover, in agreement with the proposal of Ahmed and Boudreau (2024),^18^ the C domains modulating the incorporation of dehydrobutyrine may act as dehydrating condensation domains (C_modAA_) converting l-threonine to dehydrobutyrine.^35^ Further analyses are required to verify this assumption as neither dereplication with ‘Natural Product Domain Seeker version 2’ (NaPDoS2)^36^ nor alignments using the C_modAA_ data set from Patteson et al. (2022)^35^ led to conclusive results. However, the first C-domain of DlcH and the C-domain of DlcI share high similarities during alignments (99% query coverage, 51.97% identity, E-value of 1 × 10^–108^ using NCBIs protein blast blastp), whereas the second C-domain of DlcH differs notably (95% query coverage, 25.69% identity, *E*-value of 6 × 10^–9^ using blastp), indicating similar functionalities.^24^

While Marfey’s analysis did not unambiguously resolve the configuration of ornithine (Figure S19), we assigned the l-stereoisomer to the *N* ^δ^-OH-*N* ^δ^-acetyl-ornithine and the d-stereoisomer to the terminal, cyclic *N*-OH-Ornithine based on the presence of an epimerization domain in DlcI. Moreover, DlcHs A-domain substrate specificity for ornithine is specified as formyl-hydroxy-ornithine, whereas its counterpart of DelH is specified as l-ornithine.^18^ As we did not observe any delftichelin derivative without either *N* ^δ^-acetyl-ornithine or *N* ^δ^-formyl-ornithine, we propose that the GCN5-related *N*-acetyltransferases family (GNAT) DlcN and *N* ^δ^-hydroxyornithine transformylase DlcQ modify the amino acid ornithine. The latter further indicates that the *N*-hydroxylase DlcM is also suspected to function on substrate level.

While bioinformatic analysis of the dual-condensation domain of DlcI suggested the incorporation of d-serine, analogous to the corresponding module of DelH, Marfey’s analysis revealed exclusively l-serine (Figure S20), suggesting that the relevant subdomain of DlcI may be inactive.

### Evaluation of Bioactivity

Delftichelin A (**7**) neither exhibited cytotoxic properties against HepG2 (viability of 91 ± 8% with 100 μM compound, measured in duplicate) nor antibiotic activity against tested *Acinetobacter baumannii* DSM30008, *Bacillus subtilis* DSM10, *Enterococcus faecium* ATCC51559, *Escherichia coli* Δ*acrB* (JW0451–2), *Klebsiella pneumoniae* DSM30104, *Mycobacterium tuberculosis* H37Ra, *Staphylococcus aureus* ATCC29213 or *S. aureus* Newman strains (up to 64 μM). Thus, at this stage we had to conclude that structural differences from delftibactins might render delftichelin A (**7**) inactive against a range of Gram-positive and negative bacterial strains, in contrast to its previously reported congener delftibactin A (**1**).^15^

Due to its metallophore-based structure, we then tested the affinities of delftichelin A (**7**) toward Au­(III), Cu­(II) and Fe­(III) using isothermal titration calorimetry (ITC) by direct titrations of 1 mM metal solution to 50 μM delftichelin A (**7**). The dissociation constant (*K*_D_) of delftichelin A (**7**) with Au­(III) was determined as 3.95 × 10^–6^ M ± 791 × 10^–9^ (Figure S21), while for Cu­(II), the *K*_D_ was calculated to be 1.43 × 10^–6^ M ± 217 × 10^–9^ M (Figure S22). Interestingly, the integrated heat curve and the correlated enthalpy suggests an endothermic process (Figure S22). This could possibly be dictated by a solvation effect of the copper ions.

For Fe­(III), ITC measurements yielded repeatedly *K*_D_ values in the low nanomolar range, which was expected to be a significant underrepresentation of the actual value as siderophores typically exhibit *K*_D_ of 10^–25^ M to 10^–49^ M,^17^ therefore likely exceeding the detection limit of the ITC device (∼100 μM > dissociation constant *K*_D_ > 1 nM) (Figure S23).^37^

We then conducted a displacement experiment to further narrow down the binding affinity of delftichelin A (**7**) to Fe­(III). Titration of 1 mM EDTA into a solution of 50 μM [Fe­(III)–delftichelin A] demonstrated that EDTA efficiently removes Fe­(III) from delftichelin A (Figure 6) resulting in an apparent *K*_D_ that again likely exceeds the detection limit of the ITC assay (60.8 × 10^–9^ M ± 27.9 × 10^–9^ M; Figure 6). Considering that displacement experiments reflect the difference in binding affinities of the two chelators, and assuming that the reported Fe­(III)–EDTA binding affinity of 10^–25^ M remains comparable in 50% DMSO,^38^ the upper limit of the dissociation constant for Fe­(III)–delftichelin A can be estimated to be approximately 10^–16^ M. Considering that the *K*_D_ obtained from direct titration provide the lower boundaries, these overall results suggest that the Fe­(III) binding affinity of delftichelin A lies within the subnanomolar to femtomolar range (≈1 nM to 10^–16^ M), although precise determination is limited by the sensitivity of the experimental methods. Overall, our results indicate that delftichelin A (**7**) exhibits a higher binding affinity toward Fe­(III) compared to Au­(III) and Cu­(II), suggesting that these metals are less likely to represent the primary functional targets of the metallophore.^16,18^

**Figure 6:**
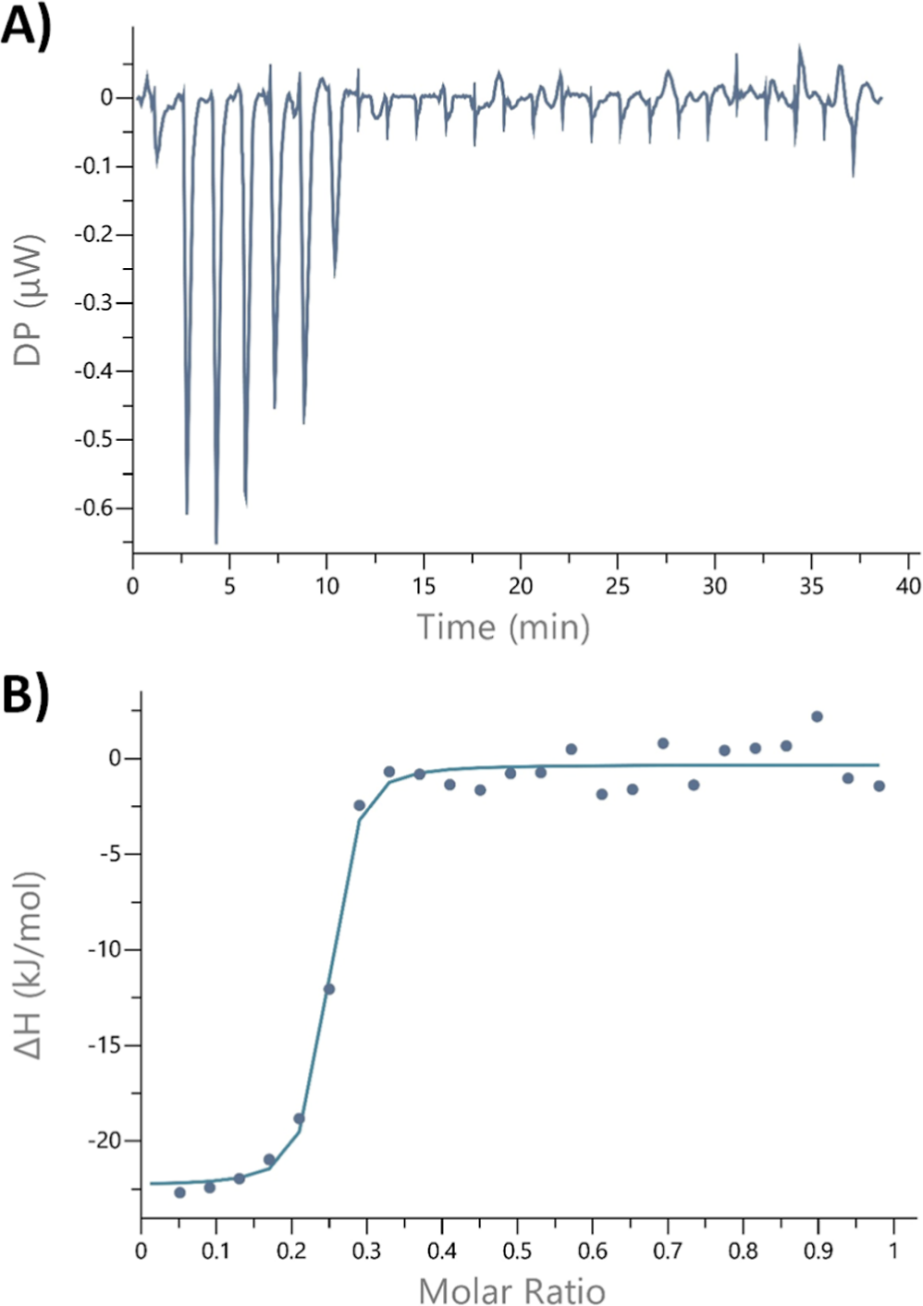
Displacement-ITC at 25 °C of 1 mM EDTA into a solution of 50 μM [Fe­(III) delftichelin A]. (A) Differential heating power over time. (B) Normalized heats of reaction (Δ*H*) against the molar ratio of Fe­(III)-EDTA binding and Fe­(III)-delftichelin A dissociation. Displayed dots depict integrated peaks, whereas the line represents the fit for the experimental data.

We also tested the stability of delftichelin A (**7**) against oxidative degradation in the presence of metal ions. For this, delftichelin A (**7**) was kept in the presence of equimolar amounts of either AuCl_3_ or CuCl_2_ at room temperature, and compound degradation measured after 24 h of incubation via UHPLC-HRMS/MS. Indeed, over time, the interaction of delftichelin A (**7**) and Au­(III) or Cu­(II) led to the detection of a previously undetected major ions (*m*/*z* 941.4201 [M + H]^+^, Table S5). Inspection of the HRMS/MS fragmentation pattern of the degradation product indicated the conversion of *N*-acetyl-ornithine to hydroxyproline (Table S5, Figure S24). This result matched with a recent report that demonstrated the function of delftibactin A (**1**) in gold ion reduction and its degradation mechanism by oxidative degradation of the hydroxyornithine.^16^ Similar findings were reported for delftibactin C (**3**).^14,27^

## Conclusion

Members of *Delftia*, such as *D. acidovorans* and *D. tsuruhatensis*, have been linked to human infections,^39^ including bacteremia,^40^ endocartitis,^41^ keratitis^42^ or pneumonia^43^ in rare cases.^44^ However, *Delftia* species have also attracted attention as effective plant growth-promoting rhizobacteria (PGPR),^45–47^ as biocontrol agent against plant pathogens,^48^ and their ability to tolerate and remediation of heavy metals in polluted environments.^49,50^ In this study, we analyzed NRPS/PKS hybrid metallophore BGCs from *Delftia* species showing homology to previously reported delftibactin-like BGCs, which led to the identification and analysis of three distinct delftibactin-encoding BGCs (*del*, *dlc, dlp*) encoded by members of the genus *Delftia*, including both pathogenic and nonpathogenic representatives.^23^ Pathway comparison of the BGCs *del*, *dlc* and *dlp* allowed to deduce major differences within the core NRPS-PKS region and the targeted isolation of delftichelin A (**7**) from *D. deserti* DSM1621, while the product of *dlp* BGC encoded by *D. deserti* KCTC42377 remains cryptic. Our metal-binding studies confirm the preference of delftichelin A (**7**) for ferric ions and its degradative interaction with Au­(III) and Cu­(II), thereby supporting the hypothesis that members of this broader compound family facilitate the growth of *Delftia* species in diverse habitats by supplying essential ferric iron while protecting cells from potentially elevated toxic metal concentrations in their surrounding environment, thus conferring a dual advantage to the producing organism.

## Experimental Section

### General Experimental Procedures

HPLC analysis was performed with Vanquish Core UHPLC system (Thermo Fisher Scientific, Waltham, MA, USA) using a Phenomenex Luna 5 μm C18 (2) 100 Å column, 250 × 10 mm column. High-resolution mass spectrometry (HRMS) measurements were performed on a Vanquish Flex UHPLC system (Kinetex C18 column (50 × 2.1 mm, particle size 1.7 μm, 100 Å, Phenomenex) using mobile phases acetonitrile and H_2_O with 0.1% formic acid, 300 μL/min flow rate, 40 °C column temperature) and an Orbitrap Exploris 120 mass spectrometer. The gradient was as follows: 0–1 min, 5% B; 1–10 min, 5–97% B; 10–12 min, 97% B; 12–13 min, 97–5% B; 13–15 min, 5% B. Mass spectra were acquired in centroid mode using a range of *m*/*z* 150–1500 and higher-energy collisional dissociation (HCD) values of 20%, 30%, and 40%. Optical rotations were measured with an Anton-Paar MD 150 instrument at 20 °C with a 100 mm path length.

### Bacterial Strains and Growth Conditions

The bacterial strains *D. acidovorans* DSM39 and *D. deserti* DSM1621 (formerly *D. acidovorans* DSM1621) were purchased from the German Collection of Microorganisms and Cell Cultures (DSMZ). Lyophilized cells were rehydrated with nutrient broth (NB, source) and spread on NB-agar medium and incubated at 30 °C. Solid media were prepared using 15 g/L agar. A single colony was transferred to 5 mL liquid NB medium and incubated overnight at 30 °C under agitation. This culture was mixed 1:1 with 50% [v/v] glycerol for cryopreservation at −80 °C. For further experiments the cells were streaked onto defined medium for siderophores (DMS), which was also used for liquid cultures afterward.^27^

### Genome Sequencing, Assembly and Bioinformatics Analyses

We anticipated the genome of *D. acidovorans* DSM39 to be similar to *D. acidovorans* SPH-1 and the genome of DSM1621 to match FDAARGOS909. Yet, to our knowledge, no genome was linked to these strains with sufficient confidence, which led us to genome sequencing *D. acidovorans* DSM39 and *D. deserti* DSM1621. In preparation for whole-genome sequencing, the *Delftia* strains were streaked onto NB-agar plates and incubated overnight. Bacterial cells were stripped of two NB plates per strain using phosphate buffered saline (PBS) and the gDNA was isolated with the Quick-DNA HMW MagBead Kit (Zymo Research, Irvine, CA, USA).

Whole-genome sequencing was performed using a Minion R10.4 flow cell (Oxford Nanopore Technologies, Oxford, UK; https://nanoporetech.com/), the MinION Mk1B sequencing device and MinKNOW (v24.02.16). Library preparation was carried out using the Native Barcoding Kit 24 V14 (SQK-NBD114.24) following the native barcoding genomic DNA protocol. The raw sequences were basecalled with “super accuracy” mode and demultiplexed with Dorado (Oxford Nanopore Technologies, Oxford, UK, v0.8). For sequence quality assessment pycoQC (v2.5.2) was used.^51^ The genomes were assembled with Hybracter (v0.9.0) specifying the --auto and --no_medaka flags to ensure high assembly quality.^52^ Afterward, genome assembly quality was assessed with Checkm2 (v1.0.2) and taxonomic classification carried out with the Genome Taxonomy Database Toolkit (GTDB-Tk, v2.4.0).^53,54^

Subsequently, all genomes were annotated with the rapid prokaryotic genome annotation (PROKKA, v1.14.5) tool and BGCs predicted with the antibiotics and secondary metabolite analysis shell (antiSMASH, v8.0).^19,55,56^ antiSMASH regions were then used to create a BGC network with Biosynthetic Gene Similarity Clustering and Prospecting Engine (BiG-SCAPE, v2.0).^20^ BGC regions with 100% similarity to delftibactin, determined by antiSMASH, were extracted and aligned using the gene cluster comparison figure generator (cLINKER, v0.0.31).^22^

### Extraction and Purification of Delftichelin A

Single colonies of *D. deserti* DSM1621 were inoculated into 5 mL DMS medium for an overnight preculture and then transferred to a 3 day-old main culture using a 1:100 [v/v] dilution factor. As an iron-replete control, citric acid monohydrate (23 nM) was added to the culture.^18^ Cultures were harvested at 4 °C and 5000 g. Reverse phase solid phase extraction (RP-SPE, 5 g CHROMABOND C18ec columns for 500 mL cultures) was used to extract delftichelins from the supernatants. Eluates were collected with stepwise with ddH_2_O, 50% and 100% acetonitrile and analyzed with the chrome azurol S (CAS) liquid assay and LC-HRMS. The CAS assay was utilized to quantify siderophore production using 105 μL of CAS solution supplemented with 4 mM 5-sulfosalicylic acid and 30 μL of 1 mg/mL extract.^57,58^

Metallophore isolation of the 50% acetonitrile fraction was achieved with a Phenomenex Luna 5 μm C18 (2) 100 Å column, 250 × 10 mm column and the Vanquish Core UHPLC system (Thermo Fisher Scientific, Waltham, MA, USA) using a column temperature of 25 °C. The following gradient was applied using ddH_2_O with 0.1% formic acid added (solvent A) and acetonitrile with 0.1% formic acid (solvent B): 0–5 min, 50%; 5–20 min, 6–18%; 20–30 min, 18–100%; with a flow rate of 3 mL/min. Delftichelin A (**7**) eluted at 18.5 min.

### Phylogenetic Analysis

Phylogenetic analysis was performed using all complete genomes from the National Center for Biotechnology Information (NCBI),^24^ excluding genomes marked for inconclusive taxonomy (but including *D. deserti* KCTC42377 despite being incomplete), totaling 34 genomes. Phylogenetic trees were constructed using the Up-to-date Bacterial Core Genes (UBCG2)^59^ pipeline visualized employing R (v4.2.2, R core Team 2024, https://www.R-project.org) and the packages Ggtree^60^ and Treeio.^61^ Average nucleotide identity (ANI) values were determined using JSpeciesWS^62^ choosing the ANIm (based on MUMmer) algorithm. DNA–DNA hybridization (DDH) values were calculated using the Genome-to-Genome Distance Calculator 3.0 (GGDC).^63,64^

### Metabolomics

Following UHPLC-HRMS acquisition, the raw spectra were submitted to MZmine.^65,66^ The noise intensity threshold for MS1 spectra was set to 1 × 10^5^ and for MS/MS spectra to 1 × 10^2.5^. During chromatogram building, the minimum peak intensity was set to 1 × 10^4^ with a 10 parts per million (ppm) *m*/*z* tolerance. Isotope peaks were grouped and shared features aligned based on 0.1 min retention time and 5 ppm *m*/*z* tolerance. Duplicate peaks were combined using the average *m*/*z* and retention time when their detection undercut 1.5 ppm and 0.04 min. The processed data was then submitted to GNPS for feature-based molecular networking using default settings (min matched fragment ions: 6, min pairs cosine: 0.7).^28,29,67^

### Oxidative Degradation of Delftichelin A by Au­(III) and Cu­(II)

Detection of the degradation product of delftichelin A (**7**) was tested mirroring the findings of delftibactin A (**1**) incubation with Au­(III). Delftichelin A (**7**) was mixed and incubated with Au­(III) and Cu­(II) separately in an equimolar (1 mM) ratio. After 24 h the mixture was measured using the UHPLC-MS/MS.

### Structure Elucidation

^1^H and ^13^C NMR spectra were either recorded on a Bruker Avance III (Ascend) 700 MHz [700 MHz (^1^H), 176 MHz (^13^C)] spectrometer equipped with a 5 mm TCI cryoprobe using standard pulse programs. Chemical shifts are given ppm and referenced against the residual dimethyl sulfoxide-d6 (DMSO-*d*_6_, 2.50 and 39.52 ppm) peak. *J* stands for coupling constants. Multiplicities are described with singlet (s), broad singlet (br s), doublet (d), doublet of a doublet (dd), quartet (q), doublet of a quartet (dq), and multiplet (m).

### Delftichelin A

Yellowish white powder; ^1^H NMR (700 MHz, DMSO-*d*_6_) and ^13^C NMR (176 MHz, DMSO-*d*_6_) are shown in Table 1; HRESIMS (positive mode) *m*/*z* 1000.4573 [M + H]^+^ (theoretical for C_41_H_66_N_11_O_18_^+^). [α]^D^_20_ = +14 (*c* = 1, DMSO-*d*_6_)

### Determination of Amino Acid Configuration

To determine the configuration of delftichelin A (**7**) amino acids, 100 μg of delftichelin A (**7**) were dissolved in 100 μL half-concentrated acid (6 M HCl and 27.5% [v/v] HI individually). The solution was incubated at 100 °C for 16 h and dried afterward. Upon drying, each sample was resuspended in 20 μL of 1 M NaHCO_3_ and mixed with 50 μL L-FDVA (1% [w/v] N-α-(2,4-dinitro-5-fluorophenyl)-l-valinamide (l-FDVA) in acetone).^33^ The mixture was incubated for 2 h at 40 °C and the reaction quenched by the addition of 10 μL 2 M HCl. Samples were diluted with acetonitrile and analyzed using UHPLC-MS.

### Isothermal Titration Calorimetry

Analysis of the metallophore’s binding affinity toward the selected metal ions Fe­(III), Au­(III) and Cu­(II) was conducted using a MicroCal PEAQ-ITC system (Malvern Panalytical, Malvern, United Kingdom). The binding affinity of delftichelin A (**7**) to the tested metals was determined by titrating 50 μM of **7** in 50% DMSO/water in the 280 μL sample cell with 1 mM metal ion solutions in the same solvent. The titration was measured over 20 × 1 μL injection steps every 90 s at 25 °C, a reference power of 10 μcal/s and a stir speed of 750 rpm. The heat change during each step was recorded, and the data evaluated by integrating the area under each peak using the MicroCal PEAQ-ITC Analysis Software (v. 1.41, Malvern Panalytical, Malvern, United Kingdom). All titrations were done in duplicate.

### Displacement-ITC

Since the binding affinity of **7** with the tested Fe­(III) ions exceed the detection limit of ITC in a direct titration, a displacement approach was used to gain further insight into the metal binding properties.^37^ First, 350 μL of a 50 μM 1:1 Fe­(III) and metallophore solution in 50% DMSO/water were prepared and incubated for 2–3 h at 4 °C to form the complex. Afterwards, the reaction cell was rinsed twice with 50% DMSO/water and then 280 μL of the complex solution were added. The titration was conducted using 1 mM EDTA in the same solvent with 25 × 0.4 μL injection steps every 90 s at 25 °C, a reference power of 10 μcal/s and a stir speed of 750 rpm. Data analysis was performed as described above.

## Supplementary Material



## Data Availability

Raw 1D and 2D NMR files, gbk files of gene cluster and NMR tables have been deposited on the Zenodo repository (10.5281/zenodo.18589817). NMR data has also been uploaded to the NP-MRD database (https://np-mrd.org/) for delftichelin A (NP0351647). The genome sequence of *D. acidovorans* DSM39 and *D. deserti* DSM1621 are available at GenBank (BioProject: PRJNA1276310, BioSample *D. acidovorans* DSM39: SAMN49042133, SRA *D. acidovorans* DSM39: SRR33981858, GenBank: CP195181; BioSample *D. deserti* DSM1621: SAMN49042134, SRA *D. deserti* DSM1621: SRR33981857). HRMS/MS data (identifier MSV000099704) has been deposited on the MassIVE repository (https://massive.ucsd.edu/).

## Notes

Additional information about *Delftia* taxonomy, comprehensive BGC information, HPLC purification, ITC results, copies of the 1D and 2D NMR spectra of compound **7**, and Marfey’s assay results (PDF)
