# Supplementary material for: Genome Mining Guided Identification of the Metallophore Delftichelin A from Delftia deserti
Source: J Nat Prod. 2026 Mar 27;89(4):1318–31. doi: 10.1021/acs.jnatprod.6c00213 (PMC13122642; doi:10.1021/acs.jnatprod.6c00213)
Supplement: Supplementary file 1 [file np6c00213_si_001.pdf]

Supporting Information

Genome Mining Guided Identification of the Metallophore Delftichelin A from *Delftia deserti*

Martinus de Kruijff[1,2], *Lukas Hiller*[1,2,3], *Joy Birkelbach*[1], *Tanya Decker*[1], *Sebastian Götze*[1], *Rebecca Kochems*[1], *Rolf Müller*[1,2,3], *Anna K. H. Hirsch*[1,2,3], *Christine Beemelmanns*[1,2,3]*

[1] Helmholtz Institute for Pharmaceutical Research Saarland (HIPS) - Helmholtz Centre for Infection Research (HZI), 66123 Saarbrücken, Germany

[2] Department of Pharmacy, Saarbrücken, 66123 Saarbrücken, Germany

[3] Faculty of Medicine, Saarland University, Saarbrücken, 66123 Saarbrücken, Germany

*Corresponding author: christine.beemelmanns@helmholtz-hips.de

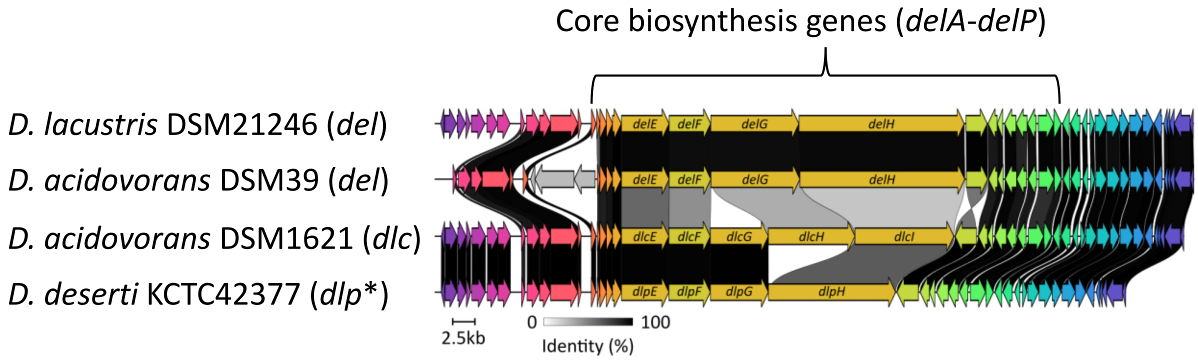

Figure S1. cLINKER comparison of core biosynthetic core genes encoding for delftibactin and delftichelin and surrounding 35 kbp. Surrounding the core biosynthesis genes, genes within the 17.5 kbp upstream and 15 kbp downstream are shown. The product of *dlp** of *D. deserti* KCTC42377 is unknown and a three-letter code was instituted. Gene similarities are indicated by links depicted with a gradient from 0% (white) to 100% (black) identity. Only links for genes with over 30% identity are shown. Gene colors show the similarities between BGCs and grey indicates similarities below 30% identity. *D. acidovorans* SPH-1 was omitted due to its almost identical BGC to *D. lacustris* DSM21246.

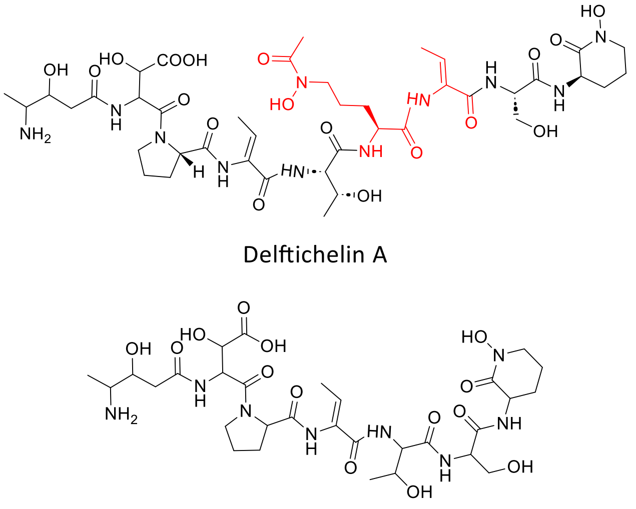

Delftichelin A

Delftipeptin

Figure S2. Comparison of delftichelin A from *Delftia deserti* DSM1621 and the putative siderophore delftipeptin from *Delftia deserti* KCTC42377. Based on bioinformatic predictions, the N-formyl ornithine and the subsequent dehydrobutyrine (highlighted in red) are anticipated to be missing in delftipeptin.

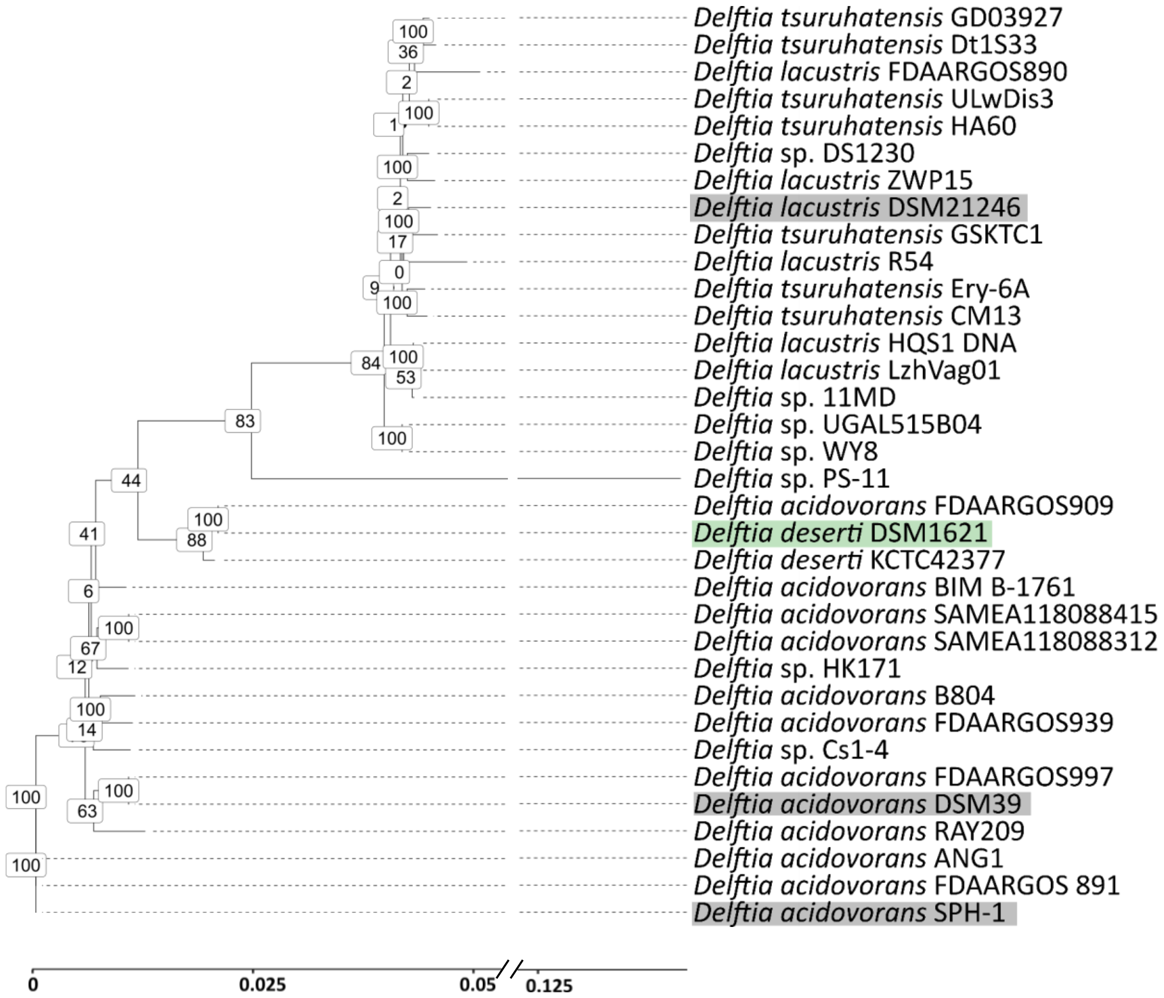

Figure S3. Phylogenetic tree of members of the genus *Delftia*. Unrooted phylogenetic tree inferred using a concatenated alignment of 81 core genes. Percentage of gene support indices (GIs), representing statistical confidence, are given at branching points. Delftichelin A producer *D. deserti* DSM1621 was highlighted in green and known delftibactin producers used for BGC analysis are highlighted in grey.

Table S1. Metabolomic potential of *Delftia* strains subdivided in BiG-SCAPE classes.

Strain	NRPS/PKS	PKS	RiPP	terpene	other	Total	Genome Accession
Delftia deserti DSM1621	3	1	1	2	3	10	This study
Delftia deserti KCTC42377	3	1	1	2	2	9	JBHSIH010000001.1
Delftia acidovorans DSM39	2		1	2	1	6	This study
Delftia acidovorans SPH-1	1		1	2	1	5	NC_010002.1
Delftia lacustris DSM21246	1	1	1	2	1	6	NZ_CP141274.1
Delftia tsuruhatensis ULwDis3	1	1	1	1	1	5	NZ_CP118775.1

Table S2. Detailed metabolomic potential of *Delftia* strains subdivided in BiG-SCAPE classes.

Strain	arylpolyene	betalactone	Butyro-lactone	NRPS/PKS-metallophore	NRPS/PKS	resorcinol	RiPP-like	terpene	terpene-precursor	Total
Delftia deserti DSM1621	1	1	1	1	2	1	1	1	1	10
Delftia deserti KCTC42377	1	1		1	2	1	1	1	1	9
Delftia acidovorans DSM39				1	1	1	1	1	1	6
Delftia acidovorans SPH-1				1		1	1	1	1	5
Delftia lacustris DSM21246	1			1		1	1	1	1	6
Delftia tsuruhatensis ULwDis3				1	1	1	1	1		5

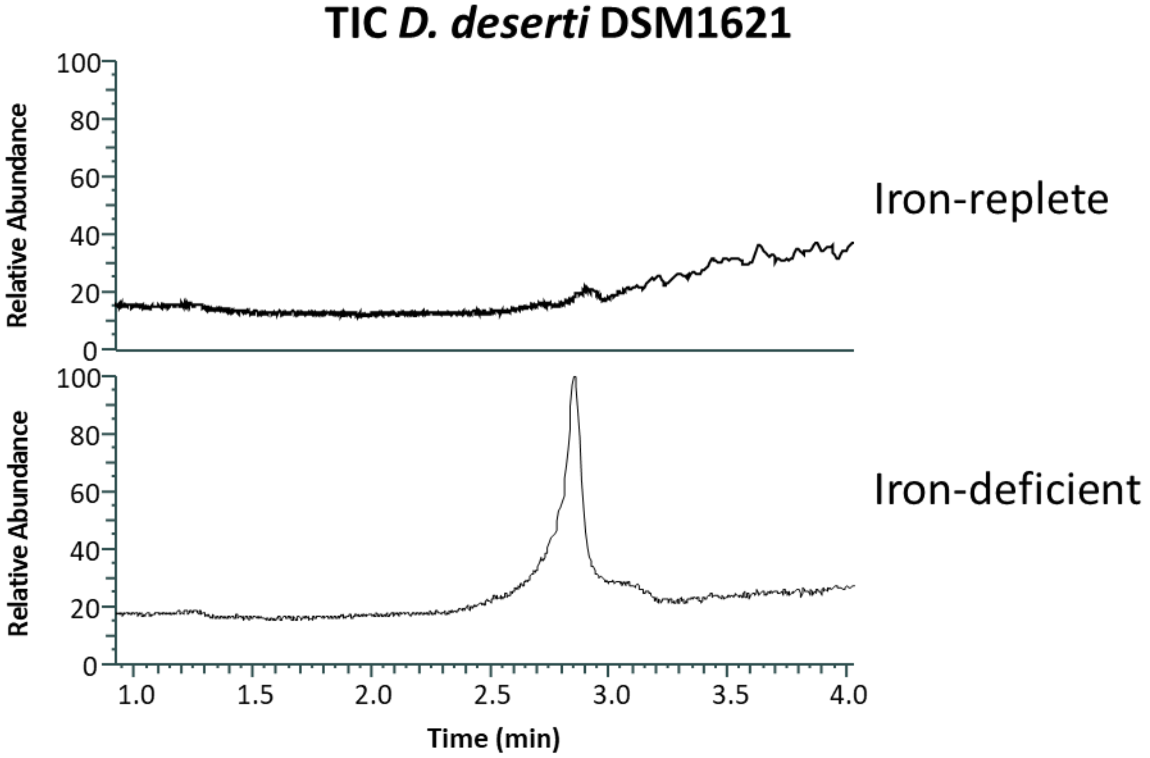

Figure S4. Total ion chromatogram (TIC) of *D. deserti* DSM1621 50% ACN crude extracts of iron-replete and iron-deficient conditions. TICs were normalized on a global scale (to relative abundance) and smoothed using the 'Moving Mean' algorithm integrated into Thermo Scientific Freestyle.

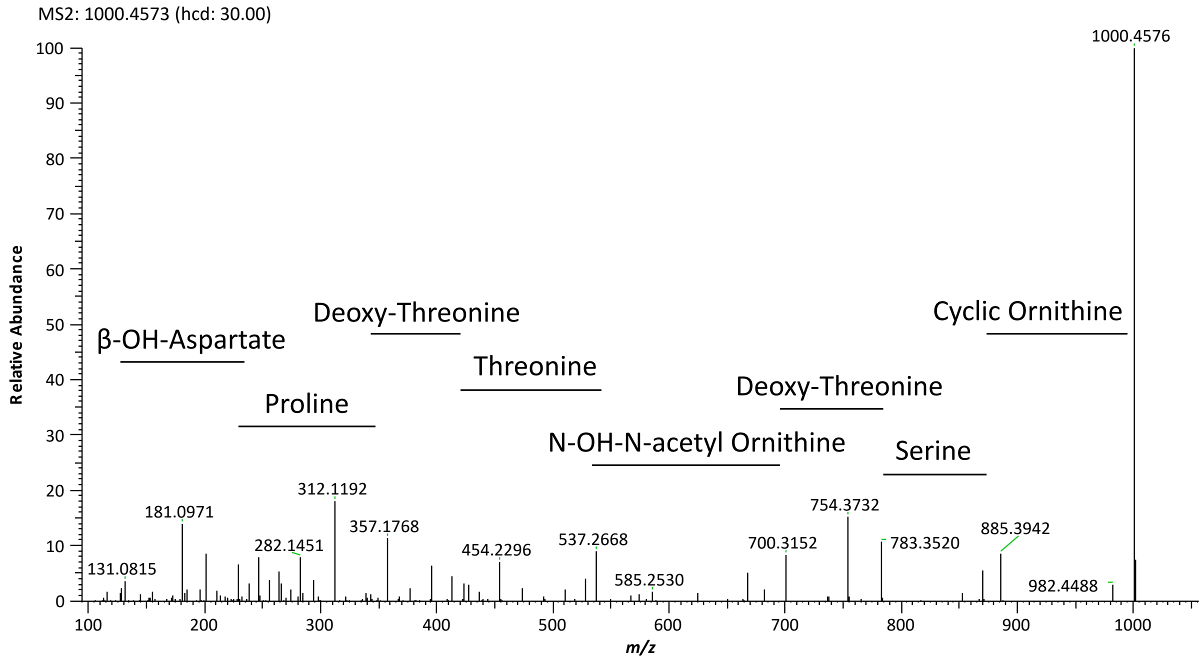

Figure S5. HRMS/MS fragmentation spectrum of delftichelin A (calculated *m/z* 1000.4582 $[M + H]^+$).

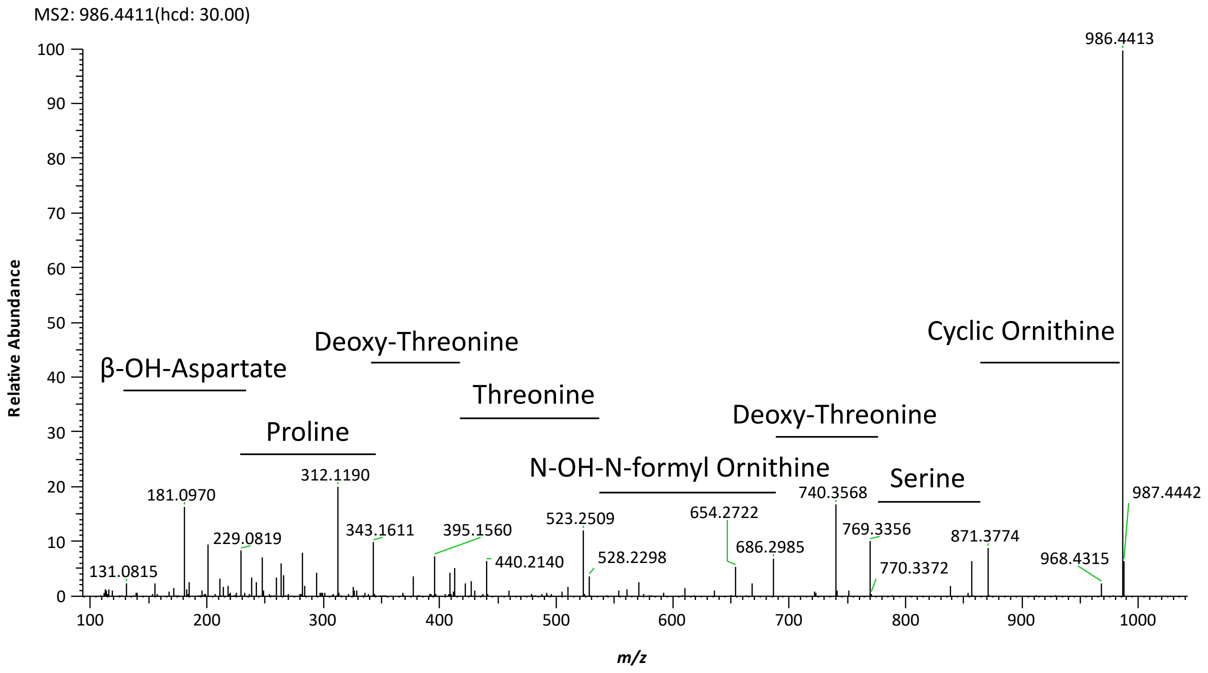

Figure S6. HRMS/MS fragmentation spectrum of delftichelin B (calculated *m/z* 986.4426 $[M + H]^+$).

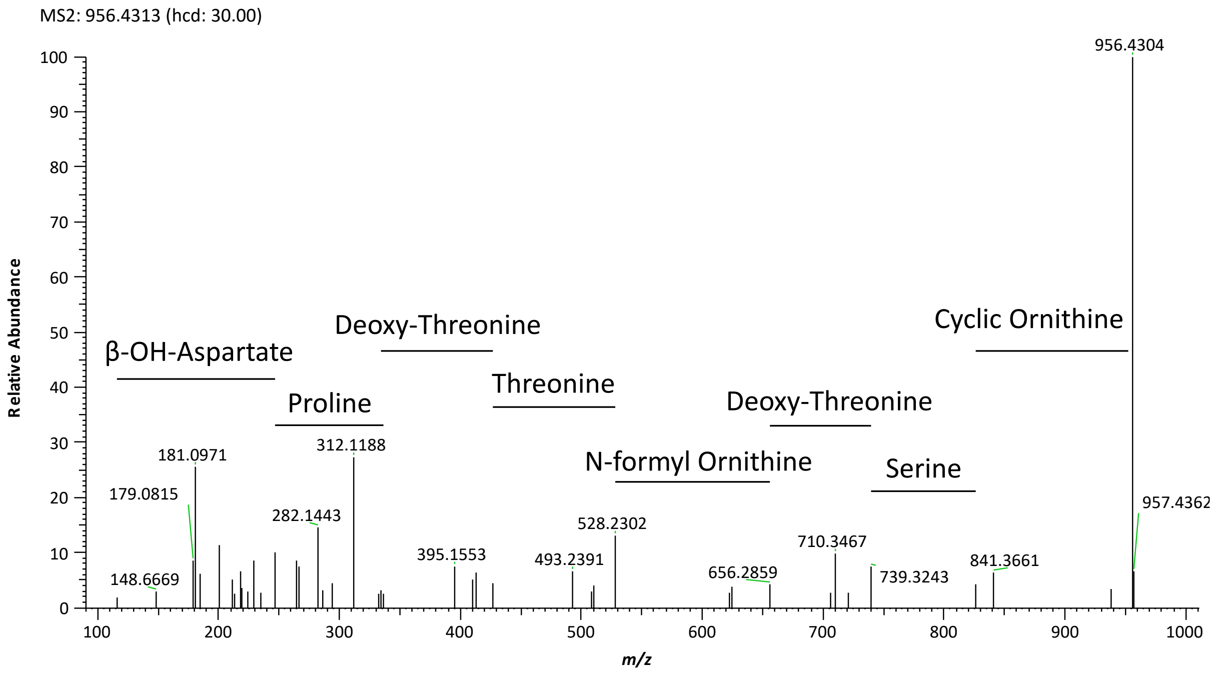

Figure S7. HRMS/MS fragmentation spectrum of delftichelin C (calculated *m/z* 956.4320 [M + H]$^{+}$).

Table S3. Comparison of theoretical and observed mass to charge ratios of delftichelin A and delftichelin B.

**Delftichelin A**				**Delftichelin B**			
Ion	Theoretical *m/z*	Observed *m/z*	Δppm	Ion	Theoretical *m/z*	Observed *m/z*	Δppm
	1000.4582	1000.4572	-0.9516		986.4426	986.4413	-1.2720
b_8	870.3845	870.3832	-1.5378	b_8	856.3689	856.3666	-2.7125
b_7	783.3525	783.3513	-1.4852	b_7	769.3368	769.3356	-1.5178
b_6	700.3154	700.3145	-1.3431	b_6	686.2997	686.2985	-1.8040
b_5	528.2306	528.2296	-1.8083	b_5	528.2306	528.2298	-1.5194
b_4	427.1829	427.1820	-2.1623	b_4	427.1829	427.1814	-3.4482
b_3	344.1458	344.1449	-2.6224	b_3	344.1458	344.1458	-0.0509
b_2	247.0930	247.0927	-1.3052	b_2	247.0930	247.0925	-2.0903
b_1	116.0712	116.0705	-5.9016	b_1	116.0712	116.0704	-6.7915

Table S4. Comparison of theoretical and observed mass to charge ratios of delftichelin C.

**Delftichelin C**			
Ion	Theoretical *m/z*	Observed *m/z*	Δppm
	956.4320	956.4307	-1.3132
b_8	826.3583	826.3563	-2.3614
b_7	739.3263	739.3250	-1.7689
b_6	656.2891	656.2884	-1.0085
b_5	528.2306	528.2298	-1.4903
b_4	427.1829	427.1822	-1.5928
b_3	344.1458	344.1461	0.8357
b_2	247.0930	247.0925	-2.1668
b_1	116.0712	116.0704	-6.7209

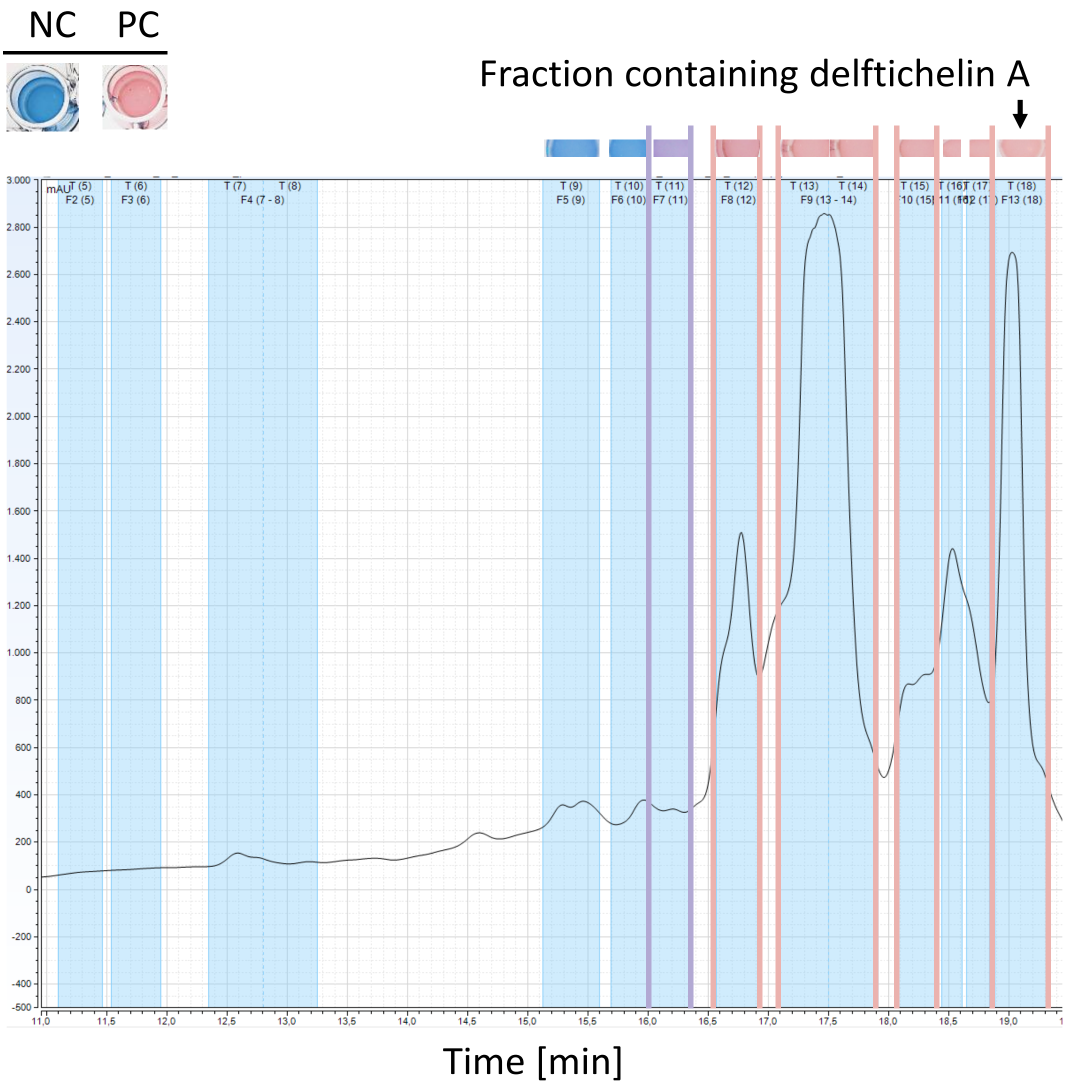

Figure S8. High-performance liquid chromatography chromatogram with the respective CAS-activity measurements. The delftichelin A containing fraction was highlighted

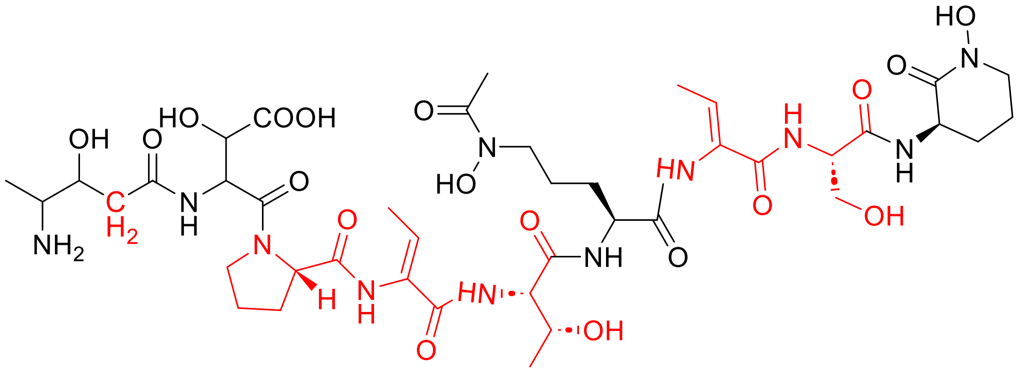

Delftichelin A

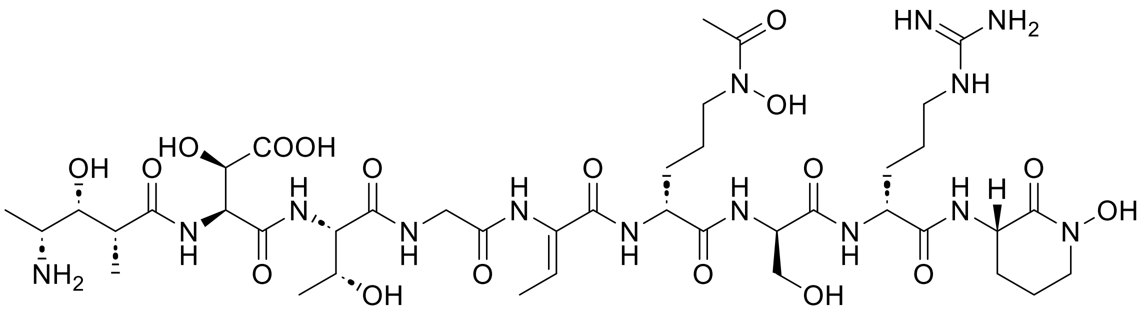

Delftibactin B

Figure S9. Comparison of delftichelin A from *Delftia deserti* DSM1621 and delftibactin B from *Delftia acidovorans* DSM39. Structural differences of delftichelin A compared to delftibactin B are highlighted in red.

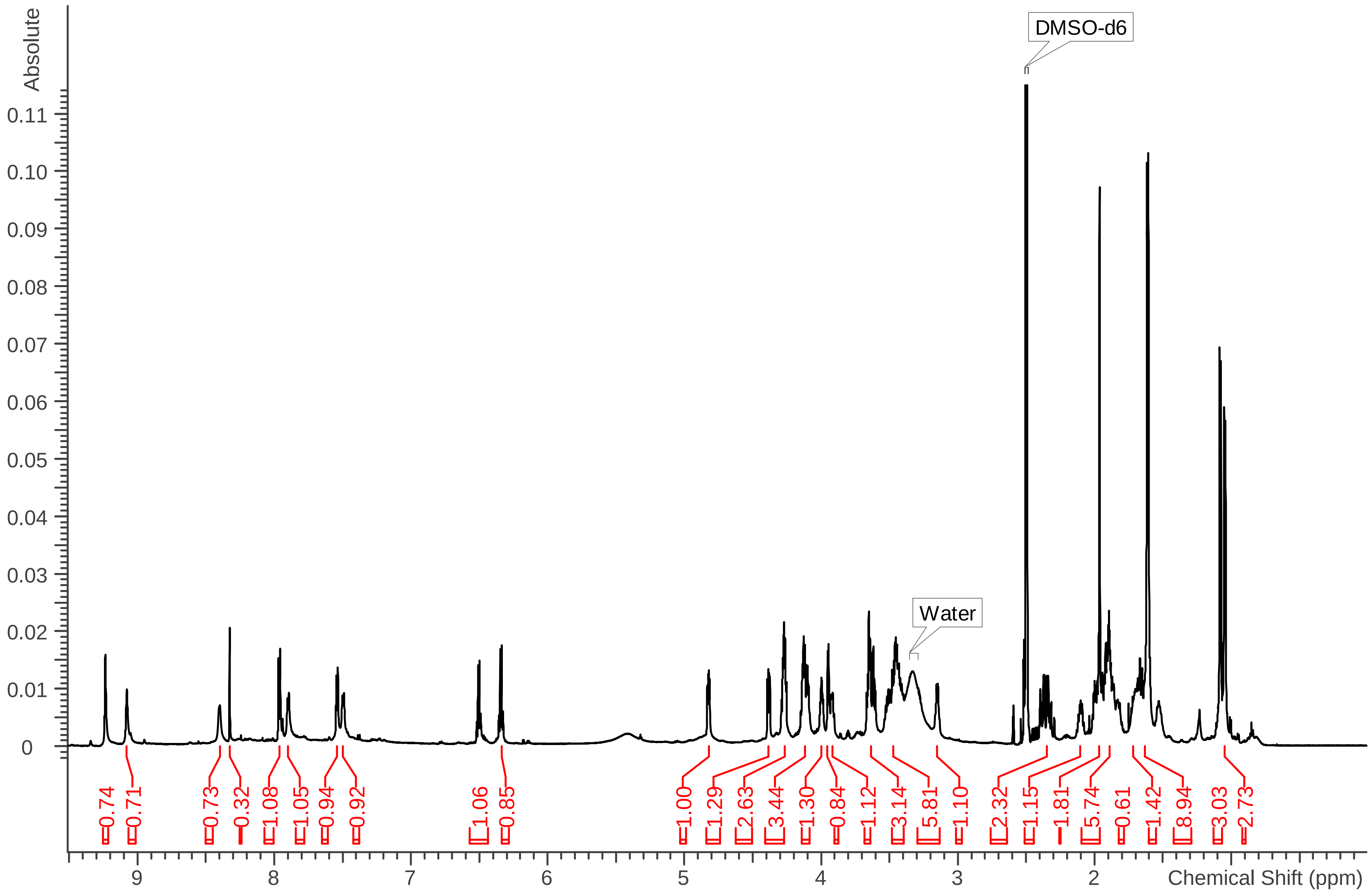

Figure S10. ^{1}H-NMR of delftichelin A in DMSO-d_6 (700 MHz)

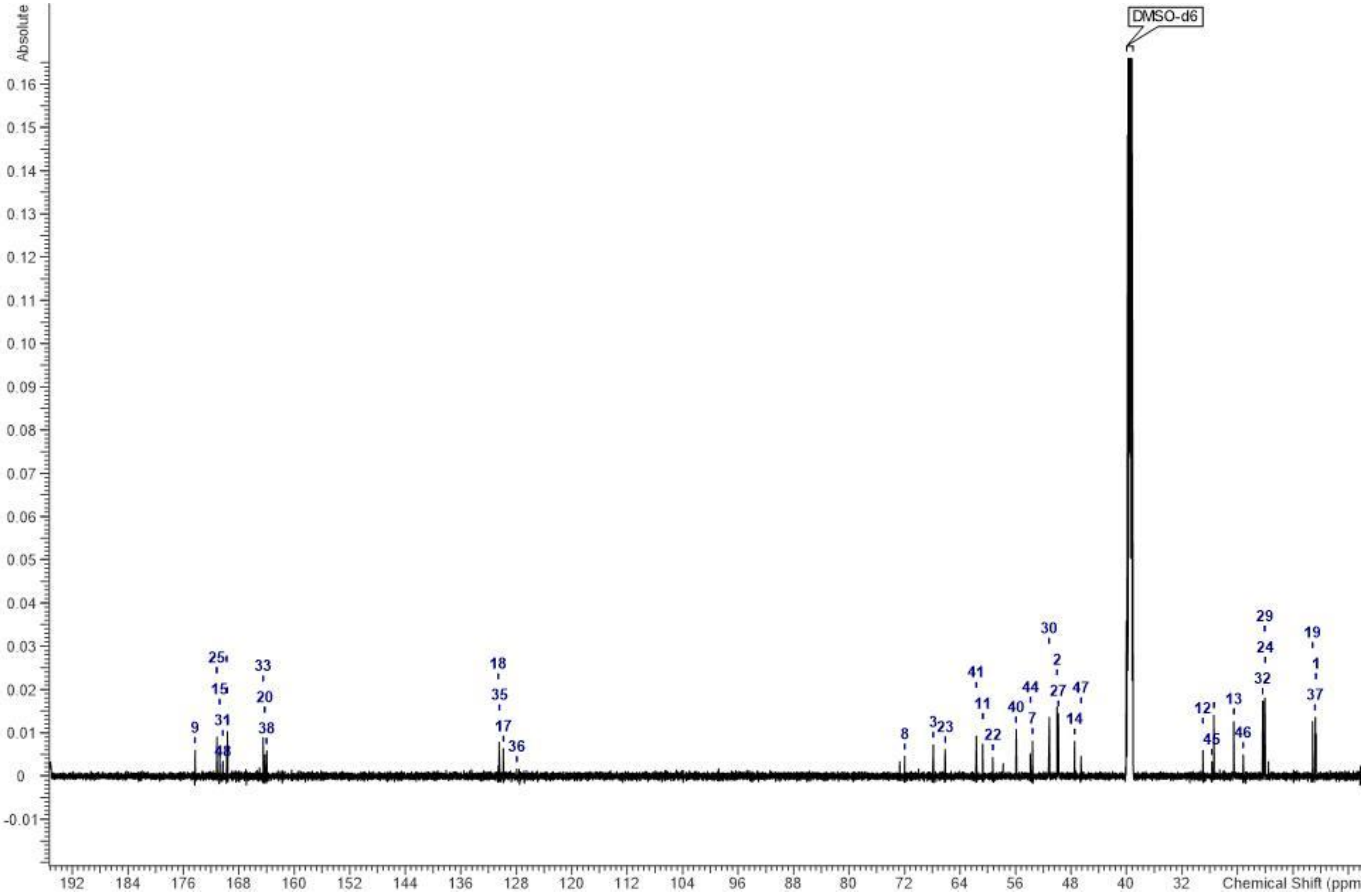

Figure S11. ^{13}C-NMR of delftichelin A in DMSO-d_6 (176 MHz)

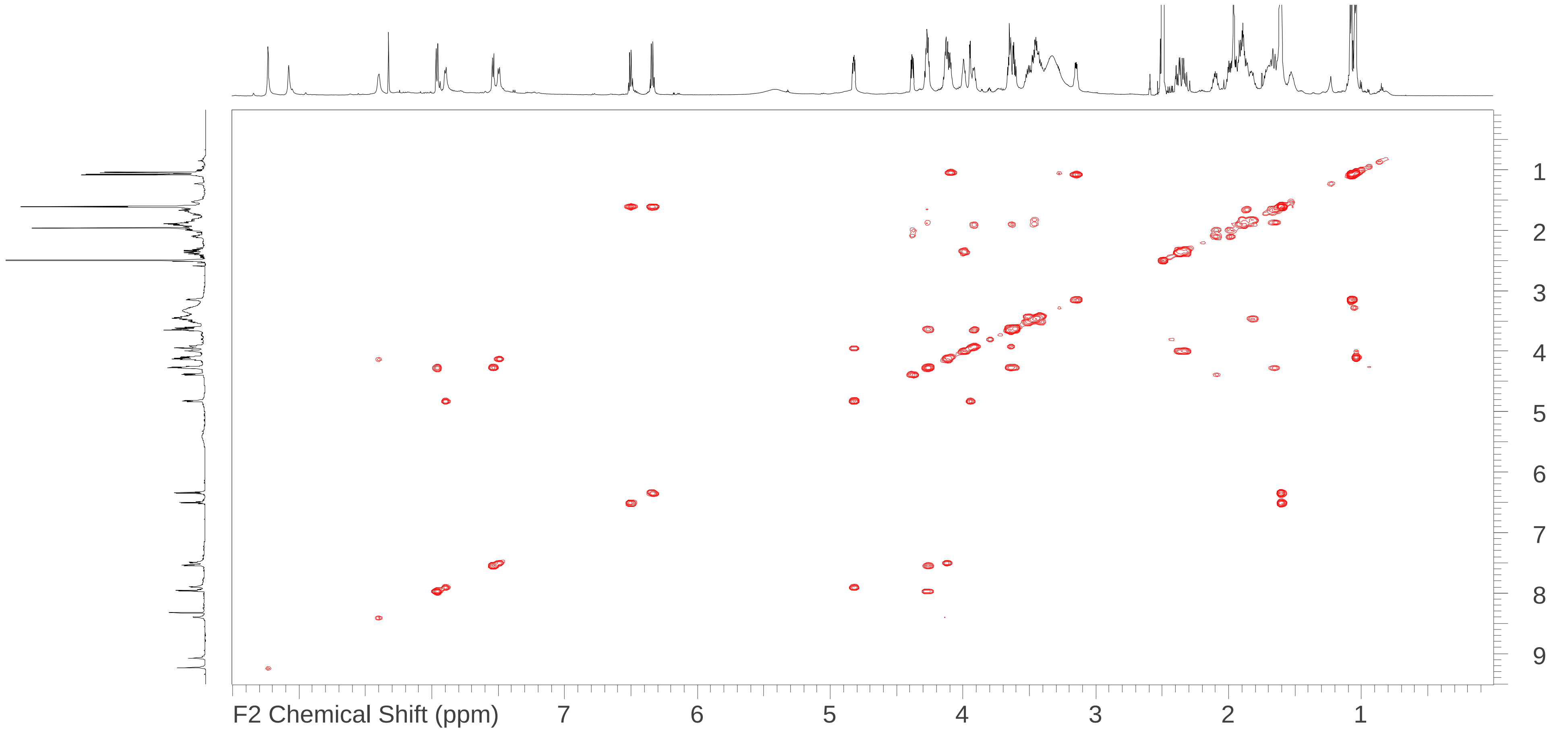

Figure S12. ^{1}H-^{1}H-COSY of delftichelin A in DMSO-d_6 (700 MHz)

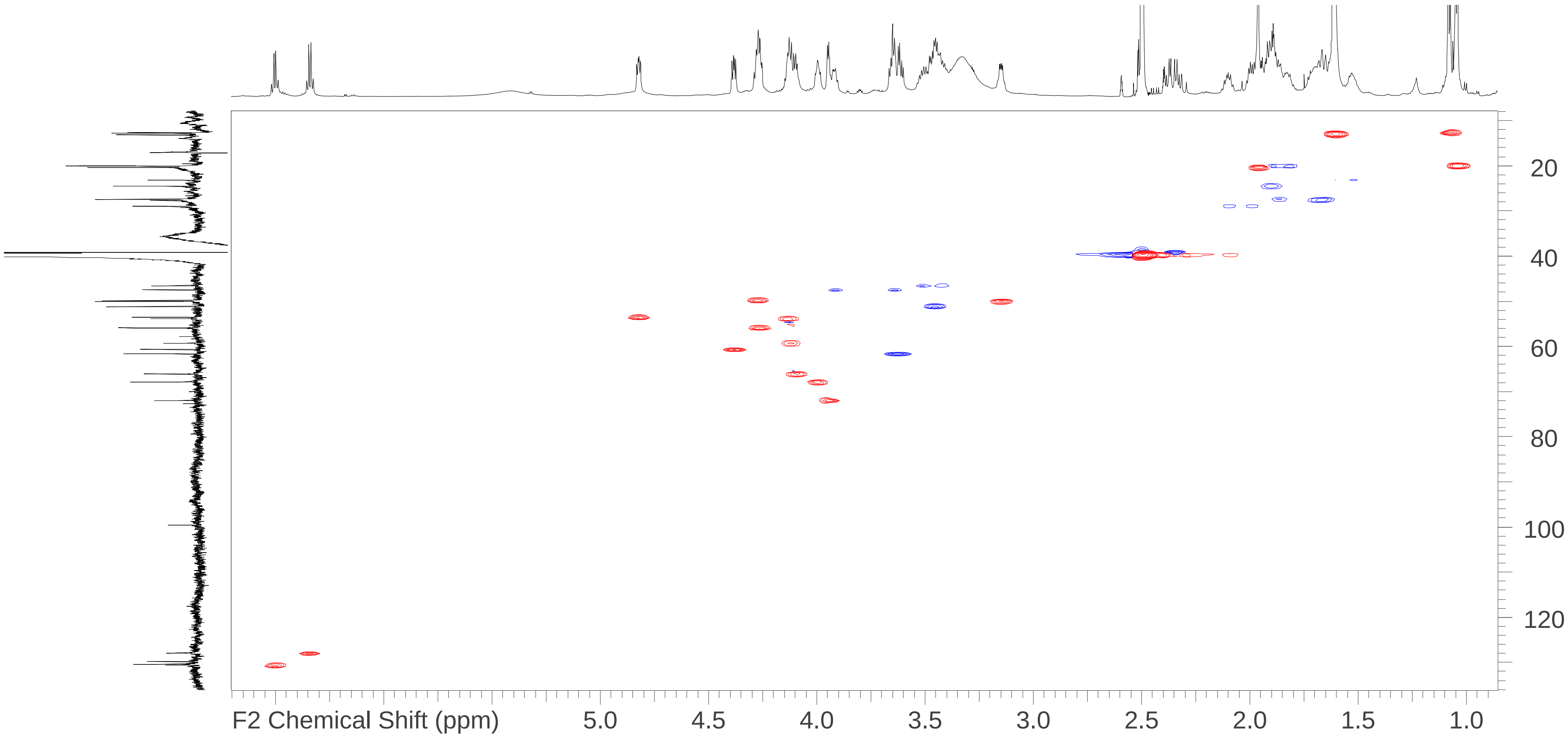

Figure S13. ^{1}H-^{13}C-HSQC of delftichelin A in DMSO-d_6 (700 MHz)

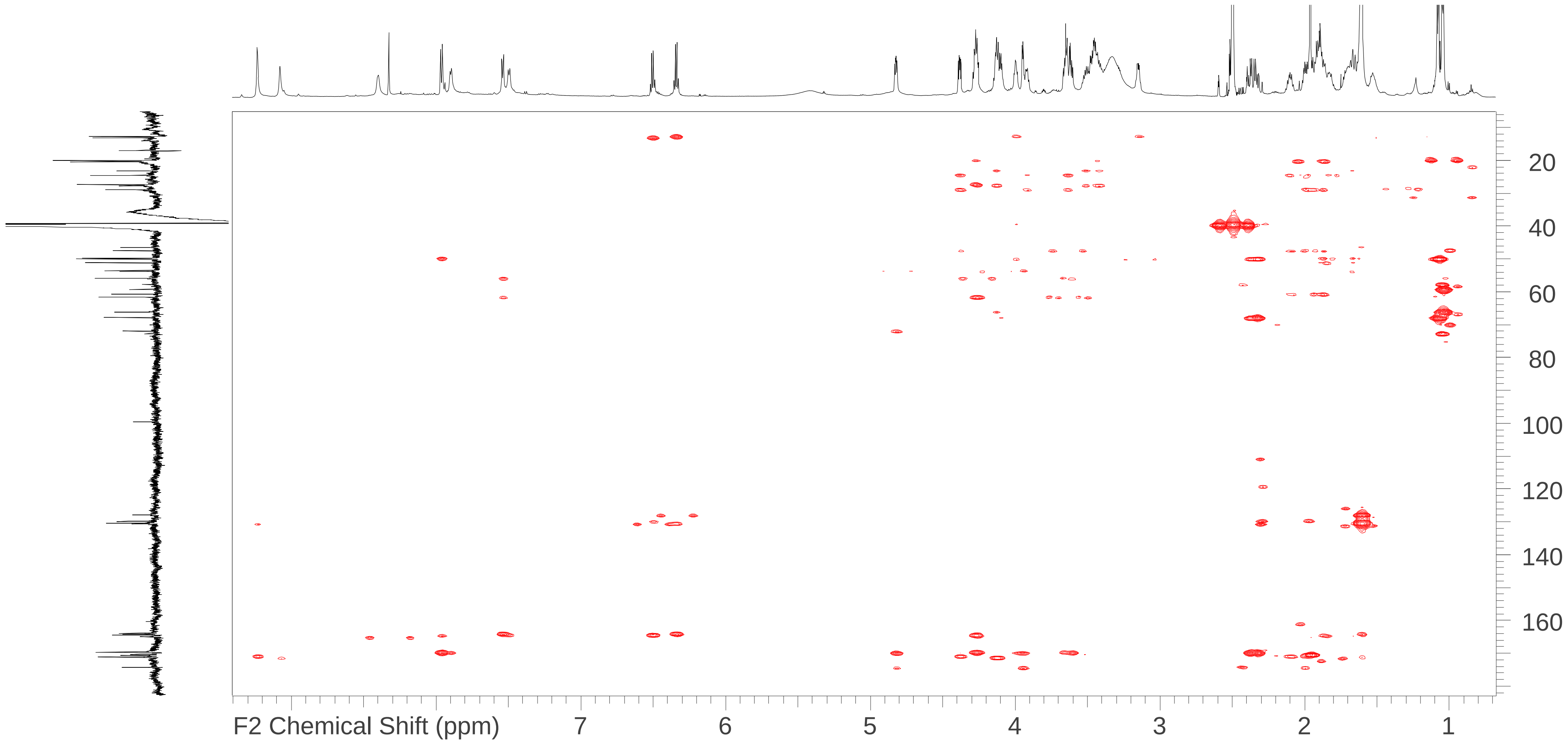

Figure S14. ^{1}H-^{13}C-HMBC of delftichelin A in DMSO-d_6 (700 MHz)

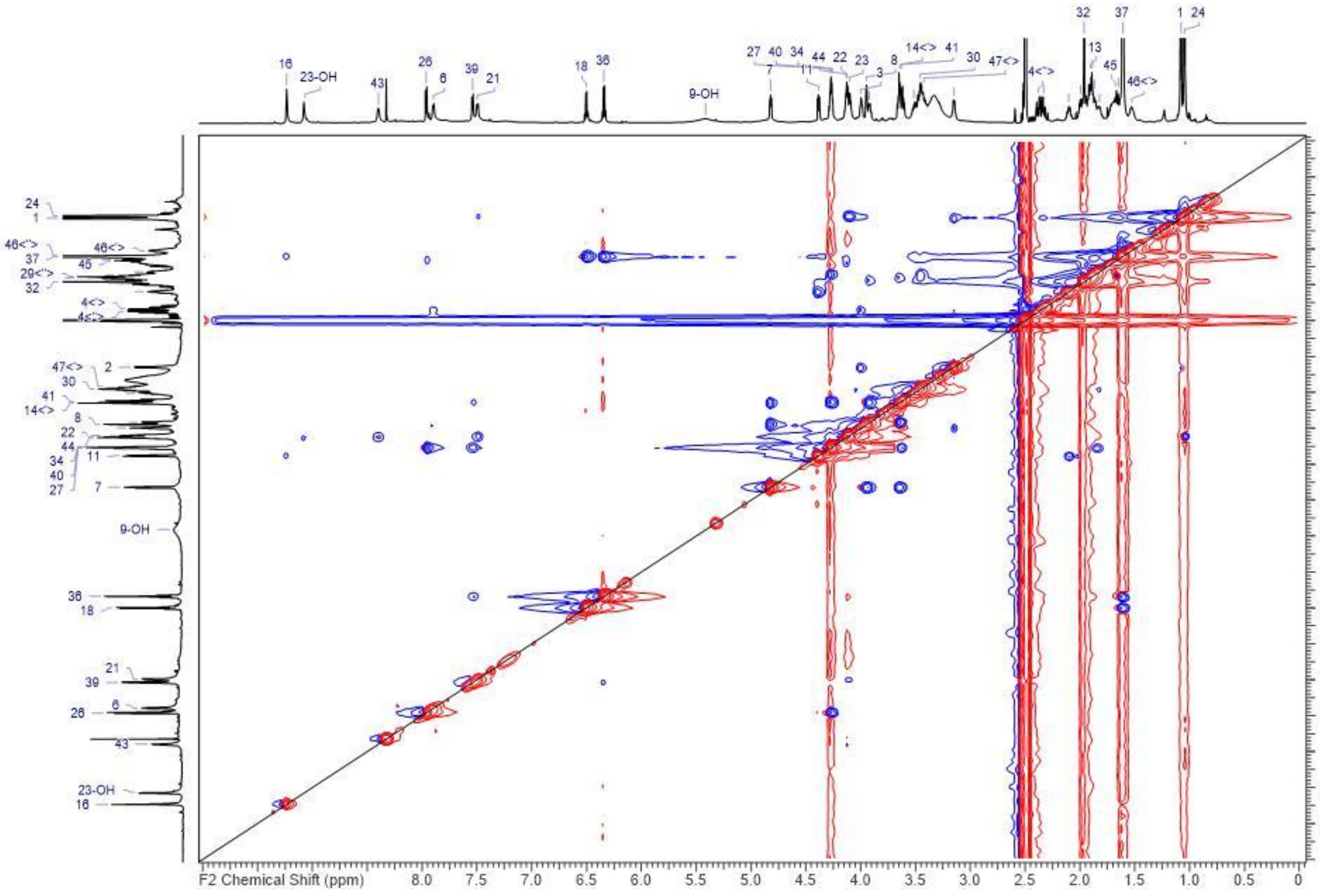

Figure S15. ^{1}H-^{1}H-ROESY of delftichelin A in DMSO-d_6 (700 MHz)

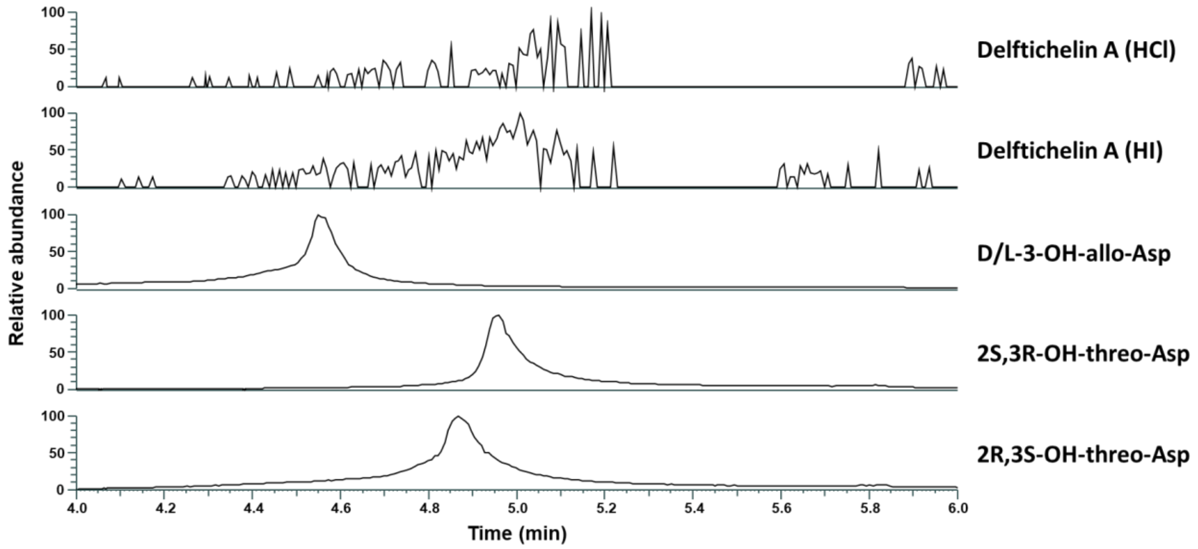

Figure S16. UHPLC-MS extracted ion chromatogram comparison of the hydrolysed and L-FDVA coupled 3-OH-aspartate (3-OH-Asp) from delftichelin A with derivatized amino acid standards. Confident configuration assignment was not possible for 3-OH-Asp of delftichelin A as no clear separation was observed.

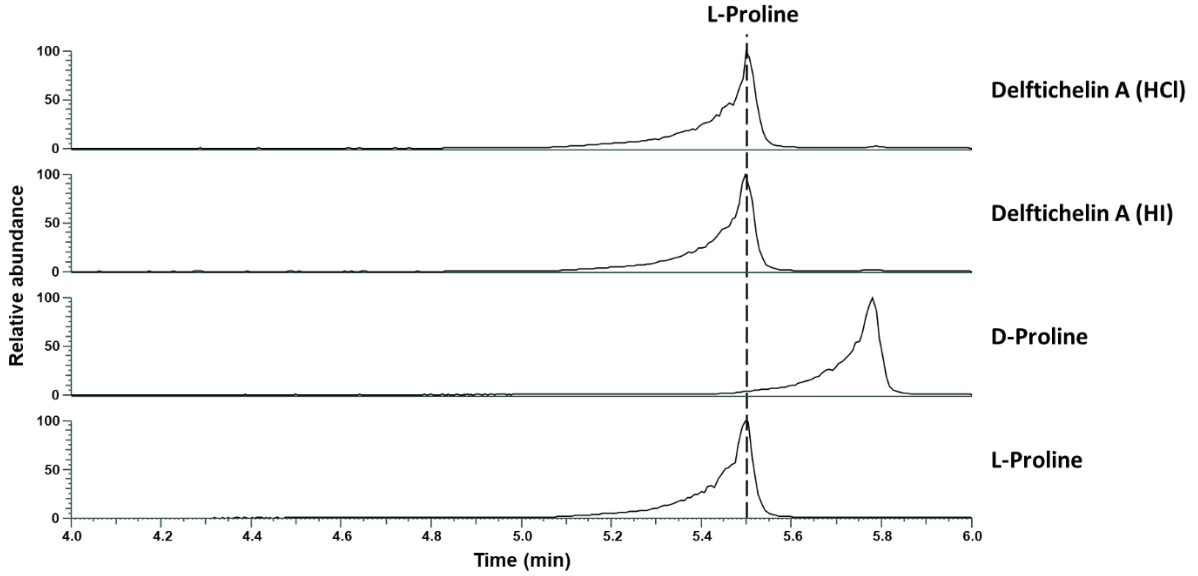

Figure S17. UHPLC-MS extracted ion chromatogram comparison of the hydrolysed and L-FDVA coupled proline from delftichelin A with derivatized amino acid standards.

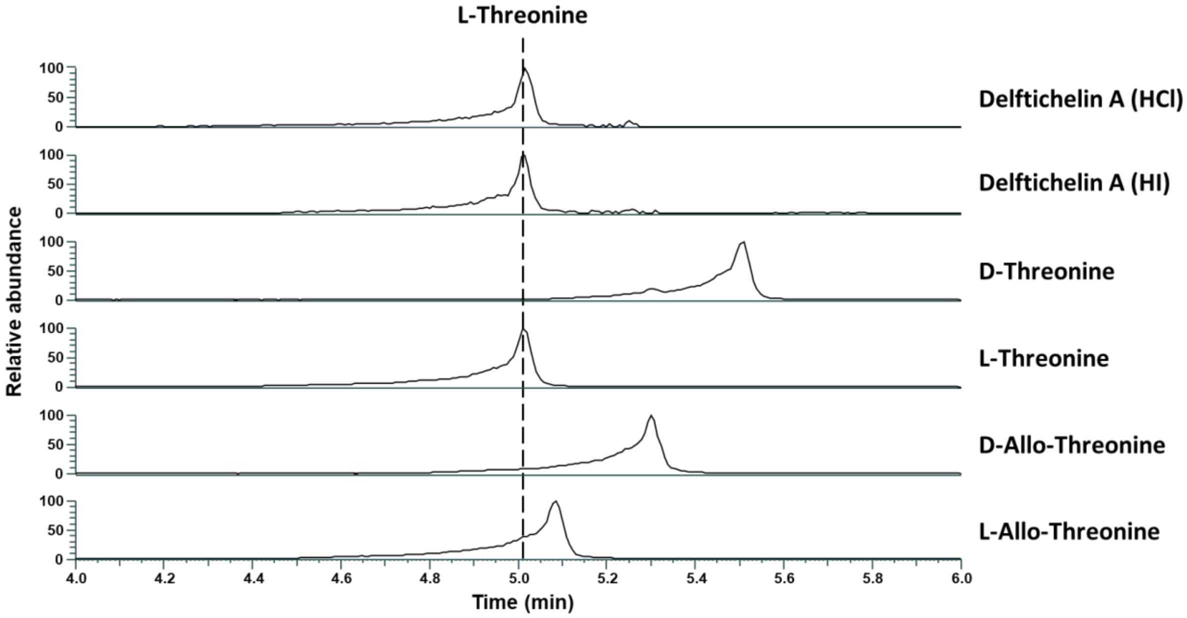

Figure S18. UHPLC-MS extracted ion chromatogram comparison of the hydrolysed and L-FDVA coupled threonine from delftichelin A with derivatized amino acid standards.

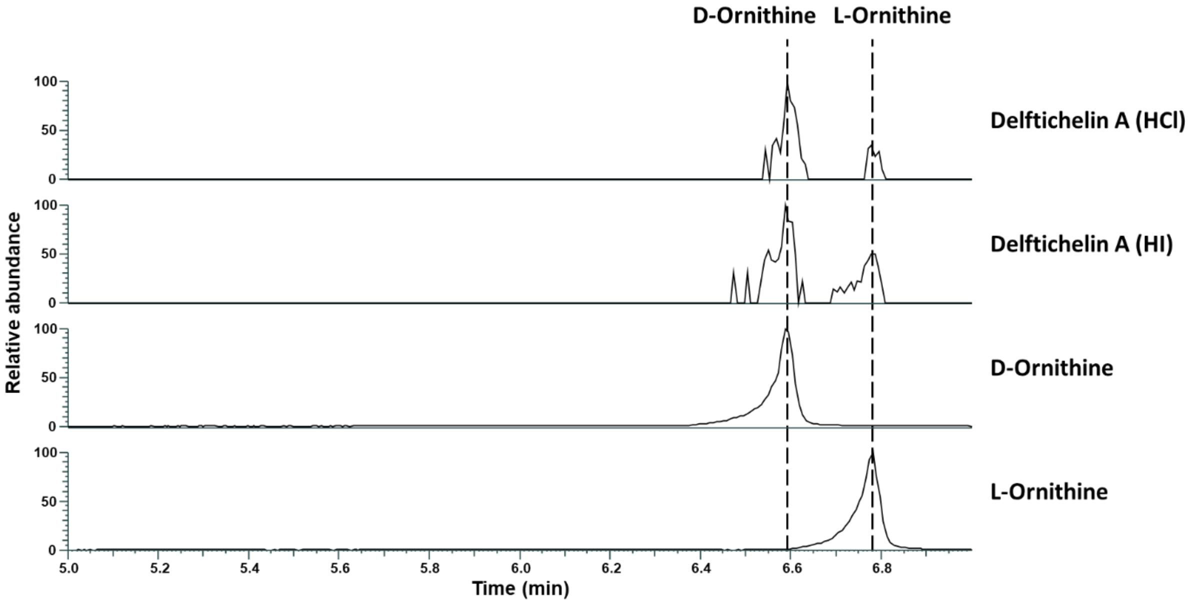

Figure S19. UHPLC-MS extracted ion chromatogram comparison of the hydrolysed and L-FDVA coupled proline from delftichelin A with derivatized amino acid standards.

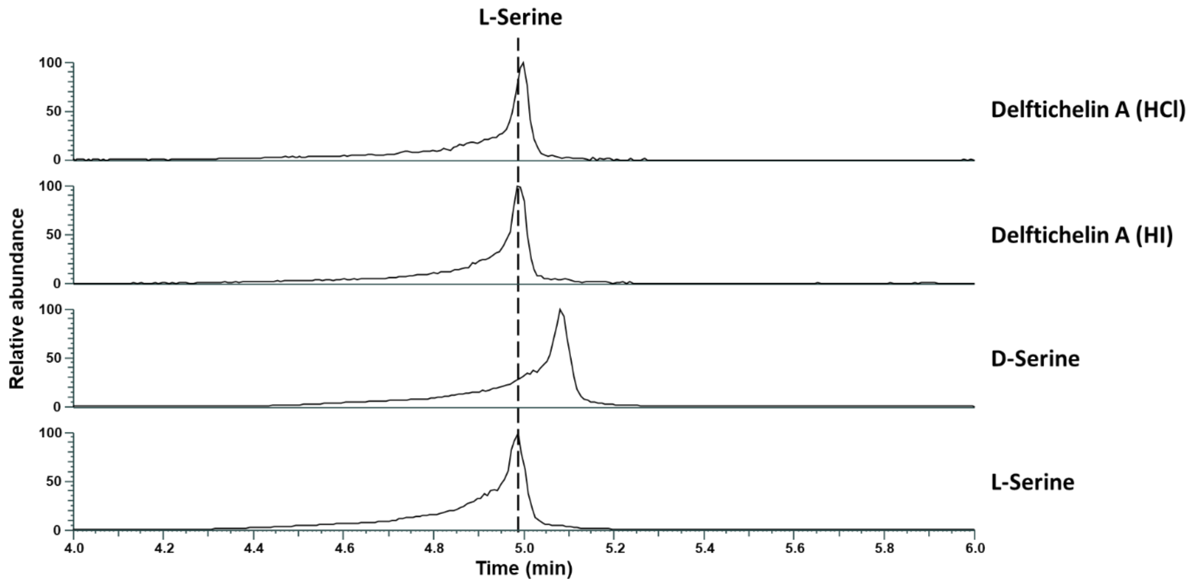

Figure S20. UHPLC-MS extracted ion chromatogram comparison of the hydrolysed and L-FDVA coupled serine from delftichelin A with derivatized amino acid standards.

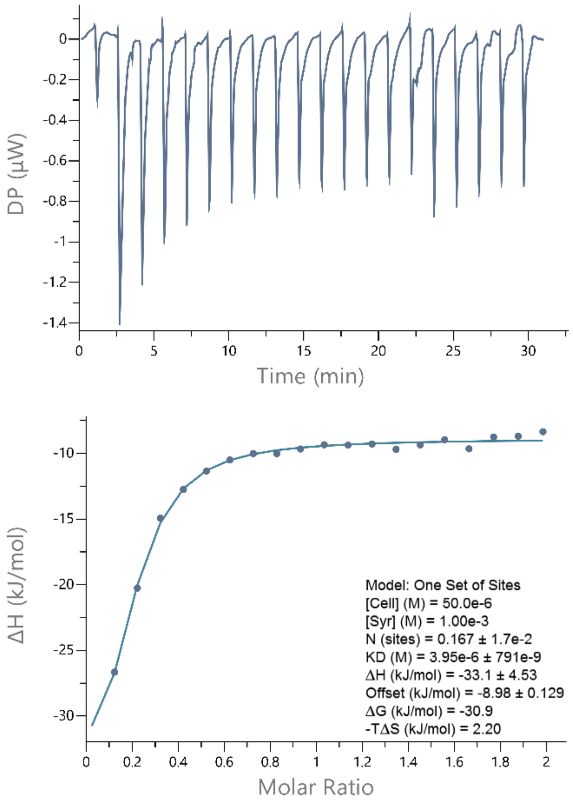

Figure S21. Direct ITC measurement of 1 mM $AuCl_3$ in 50 μM delftichelin A.

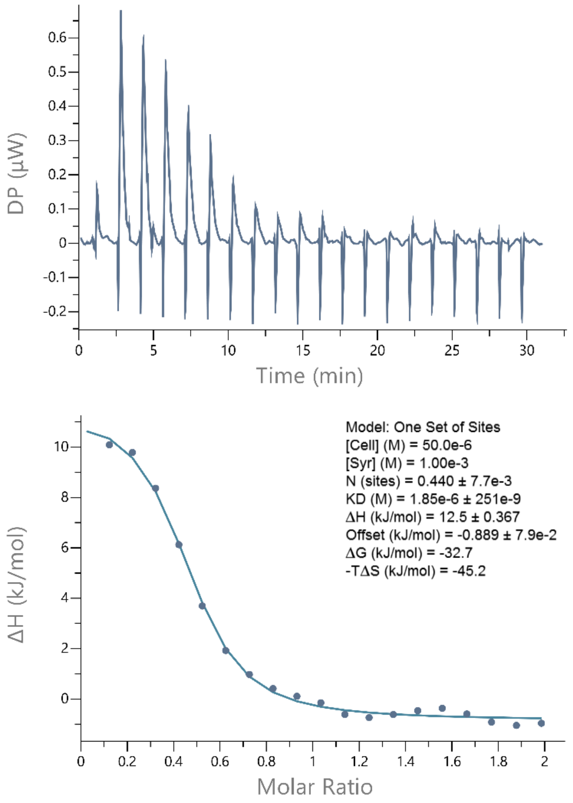

Figure S22. Direct ITC measurement of 1 mM $CuCl_2$ in 50 µM delftichelin A.

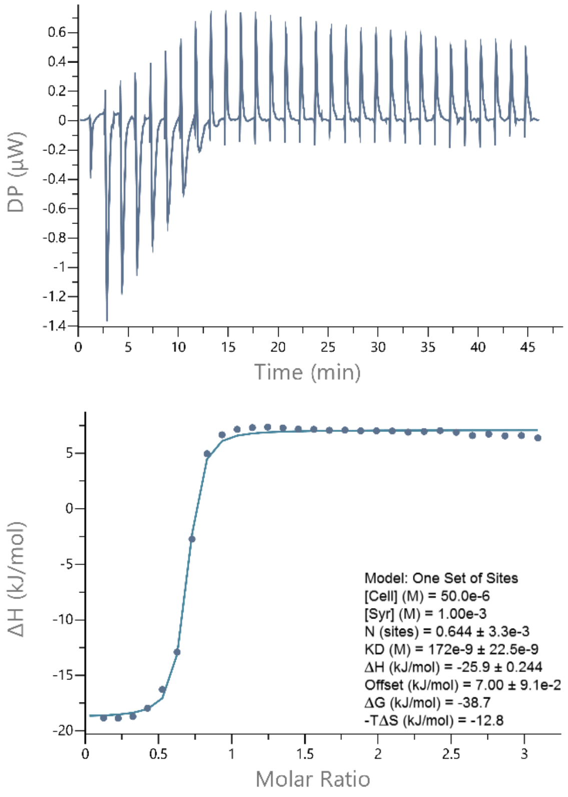

Figure S23. Direct ITC measurement of 1 mM $FeCl_3$ in 50 µM delftichelin A.

Table S5. Comparison of theoretical and observed mass to charge ratios of delftichelin A incubated with $AuCl_3$ or $CuCl_2$.

		**Delftichelin A + $AuCl_3$**				**Delftichelin A + Cu(II)**	
Ion	Theoretical *m/z*	Observed *m/z*	Δppm	Ion	Theoretical *m/z*	Observed *m/z*	Δppm
	941.4211	941.4201	-1.0700		941.4211	941.4207	-0.4774
b_8	811.3474	811.3470	-0.5083	b_8	811.3474	811.3456	-2.2082
b_7	724.3154	724.3164	1.3139	b_7	724.3154	724.3137	-2.3265
b_6	641.2782	641.2785	0.4731	b_6	641.2782	641.2740	-6.4747
b_5	528.2306	528.2299	-1.3367	b_5	528.2306	528.2301	-1.0278
b_4	427.1829	427.1819	-2.2789	b_4	427.1829	427.1819	-2.4542
b_3	344.1458	344.1448	-2.9328	b_3	344.1458	344.1450	-2.2499
b_2	247.0930	247.0925	-1.9325	b_2	247.0930	247.0925	-1.8689
b_1	116.0712	116.0704	-7.0207	b_1	116.0712	116.0705	-6.0704

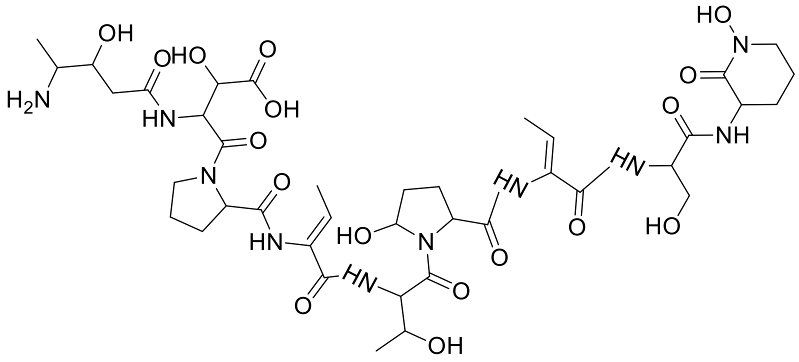

Figure S24. Oxidative degradation product of delftichelin A after incubation with $AuCl_3$ or $CuCl_2$.
